# Circular RNA expression profiling of human granulosa cells during maternal aging reveals novel transcripts associated with assisted reproductive technology outcomes

**DOI:** 10.1371/journal.pone.0177888

**Published:** 2017-06-23

**Authors:** Jing Cheng, Jia Huang, Suzhen Yuan, Su Zhou, Wei Yan, Wei Shen, Yun Chen, Xi Xia, Aiyue Luo, Da Zhu, Shixuan Wang

**Affiliations:** 1 Department of Obstetrics and Gynecology, Tongji Hospital, Tongji Medical College, Huazhong University of Science and Technology, Wuhan, Hubei, P.R.China; 2 Ultrasonic Department, Peking University Shenzhen Hospital, Shenzhen, P.R.China; 3 Reproductive Center, Peking University Shenzhen Hospital, Shenzhen, P.R.China; Peking University Third Hospital, CHINA

## Abstract

Circular RNAs (circRNAs) are a unique class of endogenous RNAs which could be used as potential diagnostic and prognostic biomarkers of many diseases. Our study aimed to investigate circRNA profiles in human granulosa cells (GCs) during maternal aging and to uncover age-related circRNA variations that potentially reflect decreased oocyte competence. CircRNAs in GCs from *in vitro* fertilization (IVF) patients with young age (YA, ≤ 30 years) and advanced age (AA, ≥ 38 years) were profiled by microarray, and validated in 20 paired samples. The correlation between circRNAs expression and clinical characteristics was analyzed in additional 80 samples. Chip-based analysis revealed 46 up-regulated and 11 down-regulated circRNAs in AA samples (fold change > 2.0). Specifically, circRNA_103829, circRNA_103827 and circRNA_104816 were validated to be up-regulated, while circRNA_101889 was down-regulated in AA samples. After adjustment for gonadotropin treatment, only circRNA_103827 and circRNA_104816 levels were positively associated with maternal age (partial r = 0.332, *P* = 0.045; partial r = 0.473, *P* = 0.003; respectively). Moreover, circRNA_103827 and circRNA_104816 expressions in GCs were negatively correlated with the number of top quality embryos (r = -0.235, *P* = 0.036; r = -0.221, *P* = 0.049; respectively). Receiver operating characteristic (ROC) curve analysis indicated that the performance of circRNA_103827 for live birth prediction reached 0.698 [0.570–0.825], with 77.2% sensitivity and 60.9% specificity (*P* = 0.006), and that of circRNA_104816 was 0.645 [0.507–0.783] (*P* = 0.043). Bioinformatics analysis revealed that both circRNAs were potentially involved in glucose metabolism, mitotic cell cycle, and ovarian steroidogenesis. Therefore, age-related up-regulation of circRNA_103827 and circRNA_104816 might be potential indicators of compromised follicular micro-environment which could be used to predict IVF prognosis, and improve female infertility management.

## Introduction

Advanced maternal age was closely associated with decreased pregnancy rates and increased spontaneous miscarriages [1]. Even seeking assisted reproductive technology (ART), only 12.2% of women aged 41–42, and 4.2% of women older than 42 years achieved live births with their own oocytes, which were remarkably lower than that of women younger than 35 years (40.1%) [2]. Due to the postponement of childbearing in modern society [3] and low success rate of ART in older women [2], age-related decline in female fertility has become a health issue urgent to be solved. Elucidating underlying molecular signatures that link maternal age and fecundity decline is particularly important for developing predictive markers and therapeutic targets for improving reproductive outcomes in aging women.

Ovarian aging or diminished ovarian function, which is generally attributed to progressive depletion of follicular reserve, decreased oocyte competence and poor granulosa cells (GCs) [4–8], has been recognized as the main driving force underlying age-related female subfertility and adverse ART outcomes [9]. Since GCs provide an essential follicular micro-environment that influences oocyte competence and early embryo development [10–12], many studies have tried to use the transcriptome of GCs as a non-invasive approach to gain insight into micro-environment surrounding oocytes and predict oocyte developmental potential [13–16]. However, there is a lack of overlap among different studies possibly owing to population heterogeneity and the instability of linear RNAs [15]. Therefore, exploring other contributors with better stability appears to be necessary.

Circular RNAs (circRNAs) are a unique class of non-coding RNAs with a stable structure formed by special loop splicing. To date, thousands of highly expressed circRNAs have been successfully identified across different eukaryotic organisms [17]. These endogenous RNAs often exhibit tissue- and developmental-phase-specific expression patterns [18, 19] and can be sorted into exosomes [20]. Compared with traditional linear RNAs, circRNAs do not have 5’ to 3’ polarity or a polyadenylated tail, but are structured as a covalently closed ring instead, thus resisting RNA exonucleases mediated degradation and maintaining a high stability [21, 22]. In addition, circRNAs contribute to post-transcriptional regulation of gene expression by binding specifically to microRNAs (miRNAs) and sequestering miRNAs to terminate suppression of their targets [23]. Increasing evidences indicate that circRNAs play important roles in aging [24, 25] and many age-related diseases, such as circ-Foxo3 in cardiac senescence [26], ciR-7 in Alzheimer's disease [27], and cir-ITCH in colorectal cancer [28]. Moreover, some circRNAs have been suggested as novel diagnostic biomarkers and potential therapeutic targets, such as hsa_circ_0005075 for prediction of hepatocellular carcinoma [29], circRNA_101222 for prediction of pre-eclampsia [30], and circRNA-CER for the treatment of osteoarthritis [31]. However, the role of circRNAs in reproduction and their contribution to ovarian aging remains to be discovered.

The aim of this study was to characterize circRNA expression profiles of GCs according to maternal age, and identify age-related changes that could potentially reflect oocyte competence, thus providing new insight into the molecular signatures of ovarian aging.

## Materials and methods

### Patients recruitment and study design

This prospective study included 126 women enrolled in ART treatment at Reproductive Medicine Center of Tongji Hospital (Wuhan, Hubei, China) between February 2015 and August 2015. Only one cycle from each patient was included in this study. Exclusion criteria were patients with polycystic ovary syndrome (PCOS), endometriosis, premature ovarian failure, preimplantation genetic screening/preimplantation genetic diagnosis cycles, and young women (≤ 30 years old) with diminished ovarian reserve (DOR, indicated by serum anti-Müllerian hormone (AMH) < 1.77ng/ml [32], or antral follicle count (AFC) < 5 on day 3 of menstrual cycle).

This study was designed as three stages. In stage 1, GCs samples from women of advanced age (AA, ≥ 38 years, n = 3) and young age (YA, ≤ 30 years, n = 3) were analyzed by a human circRNA microarray (8×15K, Arraystar Inc.). In stage 2, significantly differentially expressed circRNAs discovered in the microarray were validated in additional 20 AA and 20 YA samples by real-time quantitative reverse transcription-polymerase chain reaction (qRT-PCR). In stage 3, GC samples from another independent cohort (80 women range from 22 to 48 years old) were analyzed by qRT-PCR to further verify the association of diminished ovarian function with candidate circRNAs which were validated in stage 2. Clinical and demographic characteristics of patients were collected from electronic patient charts and summarized in Table 1, S1 and S2 Tables.

**Table 1:** CircRNA_103827 and circRNA_104816 expressions in granulosa cells according to patients’ clinical characteristics.

Variables	Mean ± SD	Min-Max	N	circRNA_103827	*P*-value	circRNA_104816	*P*-value
			(total = 80)	Mean ± SD		Mean ± SD	
Maternal age (years)	33.92 ± 7.57	22–48	-	-	-	-	-
< 37 years	-	-	40	2.49 ± 3.09	0.002**	1.89 ± 3.28	0.002**
≥ 37 years	-	-	40	6.37 ± 7.48		7.27 ± 12.86	
BMI (kg/m^2^)	21.8 ± 2.56	16.7–29.3					
< 18.5	-	-	9	2.47 ± 2.19	0.763 ^a^	1.71 ± 2.26	0.347 ^a^
18.5 ≤ BMI < 25	-	-	64	4.43 ± 6.11		5.23 ± 10.72	
BMI ≥ 25	-	-	7	6.87 ± 8.01		2.26 ± 2.90	
Baseline evaluation							
FSH (IU/l)	8.39 ± 3.95	2.65–22.25					
< 10	-	-	68	4.03 ± 5.30	0.210	4.65 ± 10.48	0.006**
≥ 10	-	-	12	6.68 ± 9.06		4.19 ± 2.68	
LH (IU/l)	4.33 ± 2.14	0.87–12.62					
≤ 4	-	-	44	4.40 ± 5.70	0.965	6.05 ± 12.55	0.681
> 4	-	-	36	4.45 ± 6.45		2.77 ± 3.61	
E2 (pg/ml)	42.52 ± 15.44	11.8–117.4					
≤ 45	-	-	49	4.74 ± 6.68	0.843	5.94 ± 11.45	0.116
> 45	-	-	31	3.94 ± 4.81		2.44 ± 5.54	
T (pg/ml)	33.16 ± 12.52	6.21–27.11					
≤ 30	-	-	35	3.97 ± 5.71	0.339	6.13 ± 11.40	0.088
> 30	-	-	45	4.78 ± 6.27		3.37 ± 8.08	
AMH (ng/ml)	3.37 ± 2.62	0.24–9.49					
< 2	-	-	34	6.50 ± 7.91	0.028*	5.61 ± 10.17	0.013*
≥ 2	-	-	46	2.89 ± 3.45		3.82 ± 9.39	
AFC (n)	10.49 ± 5.75	2–21					
< 10	-	-	44	5.94 ± 7.26	0.006**	5.73 ± 10.90	0.012*
≥ 10	-	-	36	2.58 ± 3.21		3.18 ± 7.94	
Infertility aetiology							
Male factor (n,%)	-	-	16 (20%)	3.34 ± 3.14	0.086 ^a^	2.63 ± 4.49	0.637 ^a^
Female factor (n,%)	-	-	30(37.5%)	5.77 ± 7.49		4.33 ± 7.87	
Mixed (n,%)	-	-	28 (35%)	3.58 ± 5.83		5.02 ± 11.58	
Unexplained (n,%)	-	-	6 (7.5%)	4.57 ± 3.68		8.93 ± 17.56	
Diagnosis							
Primary infertility	-	-	48 (60%)	5.34 ± 7.02	0.243	4.94 ± 9.89	0.613
Secondary infertility	-	-	32(40%)	3.06 ± 3.75		4.04 ± 9.55	

SD, standard deviation; BMI, body mass index; FSH, follicle-stimulating hormone; LH, luteinizing hormone; E2,17β-estradiol; T, testosterone; AMH, anti-Müllerian hormone; AFC, antral follicle count.

^a^ Data were analyzed by Kruskal-Wallis test, and others were analyzed by Mann-Whitney *U* test.

* *P* < 0.05;

** *P* < 0.01.

This study was approved by Institutional Review Board of Tongji Hospital (Wuhan, Hubei, China) and carried out in accordance with the principles expressed in Declaration of Helsinki. All patients who took part in the prospective observational study (NCT02294500) were informed about GCs sample collection/analysis, and written informed consents were given.

### ART procedures

Routine ovarian stimulation, oocyte retrieval, *in vitro* fertilization (IVF) / intracytoplasmic sperm injection (ICSI), embryo culture and transfer were performed at Reproductive Medicine Center of Tongji Hospital. Oocytes were considered as normally fertilized if two pronuclei and two polar bodies were observed 18–20 hours after microinjection or insemination. Embryo quality was assessed by microscopic morphological observation. Embryos that contained 7–8 regular blastomeres and less than 20% fragments on day 3 were considered as top quality. Top quality embryos were selected for transfer or freezing, whereas others were cultured up to day 5. Blastocysts on day 5 were classified according to the scoring system developed by Gardner [33]. Clinical pregnancy was confirmed by the observation of at least one gestational sac and embryonic heart activity on ultrasound examination. These patients were followed up until delivery. The detailed IVF outcomes of the participants were reported in Table 2 and S1 Table.

**Table 2:** CircRNA_103827 and circRNA_104816 expression levels in granulosa cells according to ART outcomes.

ART outcomes	Mean ± SD	N	circRNA_103827	*P*-value	circRNA_104816	*P*-value
		(total = 80)	Mean ± SD		Mean ± SD	
Retrieved COCs (n)	8.1 ± 5.6					
≤ 5	-	35	6.61 ± 7.95	0.018*	5.78 ± 11.30	0.090
> 5	-	45	2.73 ± 3.05		3.65 ± 8.28	
MII oocytes (n)	6.6 ± 4.7					
≤ 5	-	41	6.35 ± 7.58	0.012*	5.54 ± 10.56	0.016*
> 5	-	39	2.41 ± 2.51		3.58 ± 8.75	
Oocyte maturity(%)	82.3 ± 17.7					
≤ 0.8	-	28	3.33 ± 3.71	0.283	5.60 ± 11.09	0.868
> 0.8	-	52	5.02 ± 6.90		4.03 ± 8.94	
2PN (n)	4.1 ± 2.9					
≤ 4	-	50	5.66 ± 7.08	0.035*	5.09 ± 9.87	0.024*
> 4	-	30	2.38 ± 2.57		3.73 ± 9.54	
2PN (%)	56.7 ± 23.1					
≤ 0.5	-	37	5.33 ± 7.27	0.657	4.41 ± 8.87	0.49
> 0.5	-	43	3.66 ± 4.61		4.73 ± 10.48	
Embryos outcomes on Day 3						
Embryos ≥ 7 cells (n)	2.5 ± 2.3					
< 2	-	30	6.07 ± 7.72	0.045*	5.59 ± 10.70	0.077
≥ 2	-	50	3.39 ± 3.54		3.88 ± 7.32	
Embryos<20% fragmentation (n)	2.9 ± 2.6					
< 2	-	28	6.47 ± 7.91	0.034*	5.68 ± 9.91	0.009**
≥ 2	-	52	3.33 ± 4.39		3.99 ± 9.64	
Top quality embryos (n)	2.3 ± 2.0					
< 2	-	33	6.36 ± 7.43	0.009*	4.99 ± 9.26	0.037*
≥ 2	-	47	3.07 ± 4.36		4.28 ± 10.10	
Top quality embryos (%)	32.8 ± 22.7					
ratio ≤ 0.25	-	32	6.14 ± 7.43	0.047*	4.81 ± 9.70	0.265
ratio > 0.25	-	48	3.37 ± 4.70		4.02 ± 9.94	
Clinical pregnancy rate (%)	38.75					
0 ^a^	-	49	5.71 ± 6.83	0.001**	5.68 ± 10.49	0.01**
1 ^b^	-	31	2.40 ± 3.67		2.85 ± 8.18	
Live births rate (%)	28.75					
0 ^a^	-	57	5.32 ± 6.66	0.006**	5.07 ± 9.85	0.043*
1 ^b^	-	23	2.20 ± 3.09		3.37 ± 9.46	

ART, assisted reproductive technology; SD, standard deviation; COCs, cumulus oocyte complexes; MII, oocyte blocked in meiotic metaphase II; Oocyte maturity (%) = MII oocytes / COCs retrieved; 2PN = 2 pronuclear formation; 2PN (%) = 2 pronuclear fertilization rate; Top quality embryos (%) = embryos with 7–8 regular blastomeres and less than 10% fragments on day 3/ total embryos.

^a^ Failure;

^b^ Success.

All data were analyzed by Mann-Whitney *U* test.

* *P* < 0.05;

** *P* < 0.01.

### GCs isolation

On oocyte retrieval day, all follicular fluid (FF) samples from the same patient were pooled after cumulus–oocyte complexes (COCs) were isolated for conventional IVF or ICSI procedures. GCs were individually purified from FF samples with similar methods as previously described [10, 34]. Briefly, FF sample of each person was centrifuged and resuspended in phosphate-buffered saline (PBS). Then, it was slowly placed on 50% Percoll gradient and centrifuged at 400 × g for 30 min at 4°C. The cells in the middle layer were carefully collected, washed and resuspended in PBS. Next, the modified cell strainer methodology was applied to purify GCs. In order to confirm the purity of GCs, cells were stained with antibodies against CD45 (leukocyte specific antigen, not expressed by GCs) [34]. Flow cytometry revealed that the levels of CD45+ cells contamination in the samples were less than 5%. The harvested GCs were stored in TRIzol reagent (Invitrogen, Karlsruhe, Germany) at -80°C until RNA extraction.

### Total RNA extraction

All GCs samples were processed for total RNA extraction using TRIzol reagent following manufacturer’s instructions. The quantity (ng/mL) and purity (260/280 and 260/230 ratios) of RNAs were measured by a NanoDrop 2000 spectrophotometer and denaturing agarose gel electrophoresis. For RNase R digestion, 2μg RNA were incubated with 2IU RNase R (RNR-07250, Epicentre) at 37°C for 10 min followed by heat inactivation at 95°C for 3 min.

### CircRNA microarray hybridization and analysis

Sample preparation and microarray hybridization were performed according to the Arraystar’s standard protocols. Briefly, total RNAs were digested with RNase R to remove linear RNAs and enrich circRNAs. The enriched circRNAs were then amplified and transcribed into fluorescent cRNAs utilizing random primers according to Arraystar Super RNA Labeling protocol (Arraystar, Inc.). The labeled cRNAs were purified by RNeasy Mini Kit (Qiagen). As with quantification method for other RNAs [35], the concentration and specific activity of labeled cRNAs (pmol Cy3/μg cRNA) were measured by NanoDrop ND-1000. 1μg of the labeled cRNAs were hybridized onto the Arraystar Human circRNA Arrays (8x15K, Arraystar), and incubated for 17 hours at 65°C in an Agilent Hybridization Oven. The hybridized arrays were washed, fixed and scanned using the Agilent Scanner G2505C. Scanned images were imported into Agilent Feature Extraction software for raw data extraction. A series of data processing including quantile normalization were performed using R software packages. The statistical significance of the difference was estimated by t-test. CircRNAs having fold changes ≥ 2 and *P*-values < 0.05 were recognized as significantly differentially expressed. The Arraystar’s home-made computer program based on TargetScan and miRanda was applied to predict their miRNA targets and the circRNA/ miRNA interaction. The miRNA support vector regression (mirSVR) algorithm was utilized to score the efficiency of predicted miRNA targets.

### Characterization and validation of candidate circRNAs by RT-PCR

Based on fold change > 3, we selected the top 10 among 17 up-regulated circRNAs, and all 4 down-regulated circRNAs for subsequent validation. qRT-PCR was performed using Fast SYBR Green Master Mix (4385612, Thermo Fisher Scientific) according to the manufacturer’s instructions. Three paired divergent primers encompassing circRNA-specific back-splice sites were designed for each candidate circRNA. Sequences of the primers achieving a single peak in melting curve were provided in S3 Table. Relative expression levels of candidate circRNAs were normalized to glyceraldehyde-3-phosphate dehydrogenase (GAPDH) and were calculated according to 2^- ΔΔCt^ method [29, 30, 36].

In addition, the circular characteristics of differentially expressed circRNAs were assessed by two methods: testing their enrichment in poly(A)- fractions and detecting their resistance to RNase R digestion. RNA with and without poly(A) tail were fractionated using oligo-d(T)_25_-Magnetic Beads (E7490L, New England Biolabs Inc.). Then, poly(A)+ RNA, poly(A)- RNA, digested RNA and control total RNA were reversely transcribed into cDNA using the PrimeScript RT reagent kit (RR037A, TaKaRa) with random primers. Next, candidate circRNAs were amplified from cDNA by KOD Xtreme Hot Start Polymerase Kit (71975–3, Novagen). Eventually, PCR products were detected by agarose gel electrophoresis and Sanger sequencing.

### Annotation and function prediction for circRNA_103827 and circRNA_104816

Validated circRNAs (circRNA_103827 and circRNA_104816) were used as seeds to enrich circRNA-miRNA-gene networks using Arraystar's home-made miRNA target prediction software. The predicted gene functions were annotated using Gene Ontology (GO) and Kyoto Encyclopedia of Genes and Genomes (KEGG) Pathway based on Database for Annotation, Visualization and Integrated Discovery (DAVID).

### Statistical analysis

Continuous parametric data were presented as mean ± standard deviation (SD) and categorical variables were presented as numbers and percentages. Variables including maternal age, BMI, baseline evaluation, and hormonal levels at oocyte pick up in stage 2 were analyzed by Student *t* test, while other variables in stage 2 were analyzed by Chi-square test. In addition, Mann-Whitney *U* test and Kruskal-Wallis test were used to compare circRNA expression levels between two groups and multiple groups in stage 3, respectively. Spearman correlation analysis was used to investigate the correlation of circRNA expressions and clinical characteristics. The ability of circRNA levels in GCs to predict pregnancy outcomes was determined by constructing the Receiving Operator Curve (ROC) curve and calculating the area under the curve (AUC) with 95% confidence intervals (CI). The sensitivity and specificity for the optimal cut-off were calculated. Statistical tests were performed using SPSS 16.0 software. Results were considered significant when *P* < 0.05.

## Results

### Stage 1: CircRNA expression profiles in GCs according to female age

**Fig 1:**
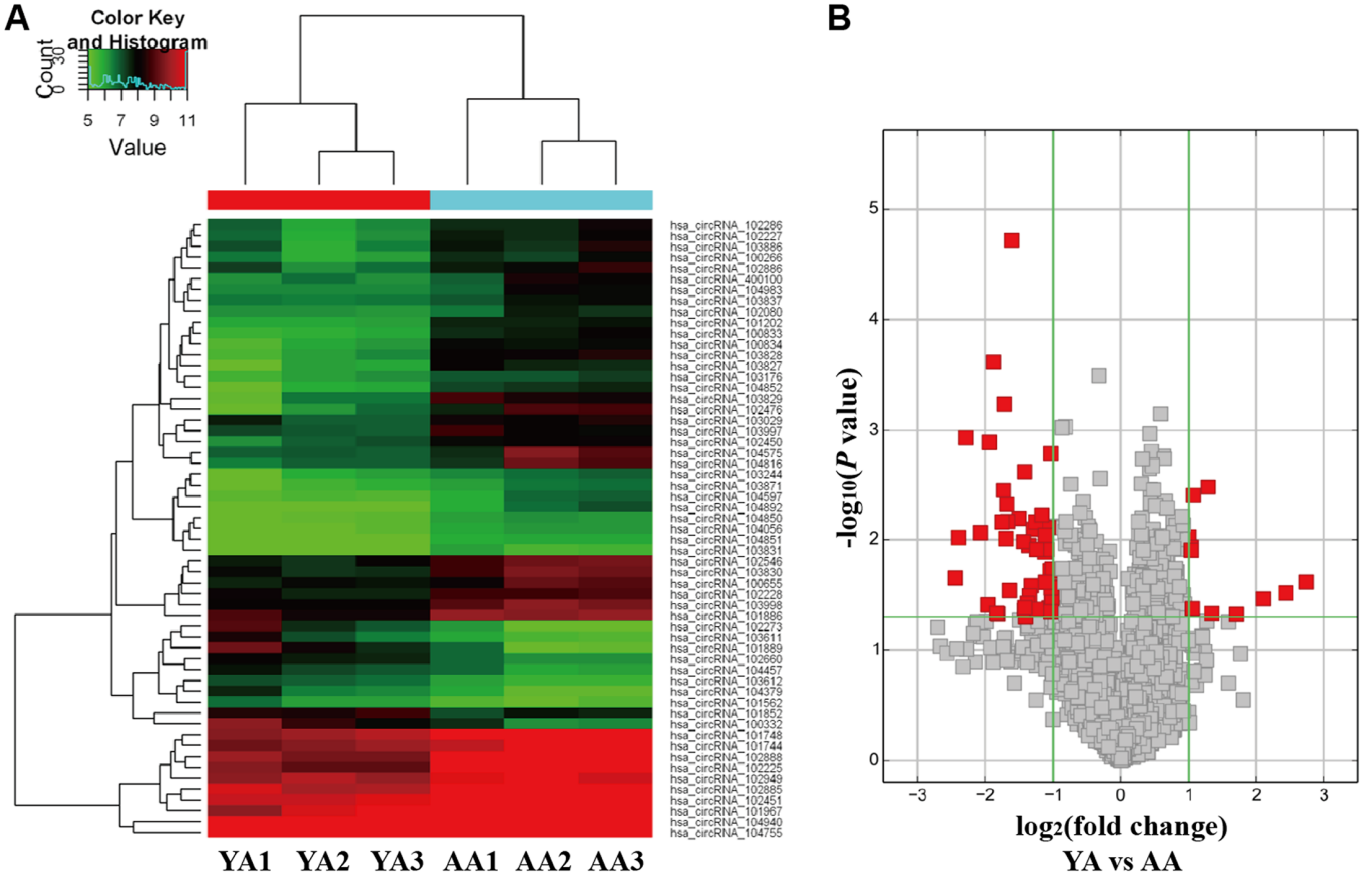
CircRNA expression profiles in GCs according to maternal age. (**A**) Hierarchical clustering analysis of differentially expressed circRNAs in GCs of three young (≤ 30 years) and three older (≥ 38 years) women. Expression values (fold change > 2.0, *P* < 0.05) were represented in different colors, indicating expression levels above and below the median expression level across all samples. (**B**) Volcano plot was constructed using fold-change and *P*-values. The vertical lines correspond to 2.0-fold up- and down-regulation between YA and AA samples, and the horizontal line represents a *P*-value. Red dots represent the differentially expressed circRNAs with statistical significance (fold change > 2.0 and *P* < 0.05). Red dots on the left represent up-regulated circRNAs in older women, while those on the right represent down-regulated circRNAs in older women. YA, women with young age (≤ 30 years); AA, women with advanced age (≥ 38 years).

GCs RNA from three YA and three AA samples were analyzed by circRNA microarray. Unsupervised hierarchical clustering of circRNA expression patterns discriminated YA from AA women obviously (Fig 1A), revealing different circRNA expression profiles in GCs during maternal aging. The variations of circRNA expression between YA and AA samples are additionally shown as a volcano plot (Fig 1B). Overall, 57 circRNAs were discovered to be significantly differentially expressed (fold change > 2.0, *P*-value < 0.05). Compared to YA samples, 46 circRNAs were up-regulated and 11 circRNAs were down-regulated in AA samples (Table 3 and S4 Table). The complete microarray data in this study was available at Gene Expression Omnibus (GEO) database under the accession number GSE97193.

**Table 3:** The top 10 significantly differentially expressed circRNAs between young and older women ranked by fold change.

circRNA	chrom	Gene Symbol	FC	*P*-value	miRNA response elements	
**Up-regulated circRNAs in older women**						
hsa_circRNA_102476	chr19	MYO9B	5.4408028	0.022099678	hsa-miR-767-3p	hsa-miR-877-3p
hsa_circRNA_103829	chr5	HMGCS1	5.2788552	0.009579475	hsa-miR-625-3p	hsa-miR-129-5p
hsa_circRNA_103828	chr5	HMGCS1	4.8616152	0.001175501	hsa-miR-411-5p	hsa-miR-625-3p
hsa_circRNA_103827	chr5	HMGCS1	4.1982568	0.008617227	hsa-miR-411-5p	hsa-miR-625-3p
hsa_circRNA_101967	chr17	FAM64A	3.9048426	0.038709954	hsa-miR-412-3p	hsa-miR-18a-3p
hsa_circRNA_100834	chr11	FADS2	3.8479172	0.001283371	hsa-miR-873-5p	hsa-miR-23b-5p
hsa_circRNA_100833	chr11	FADS2	3.6633041	0.000239541	hsa-miR-765	hsa-miR-495-3p
hsa_circRNA_104816	chr9	IARS	3.5465149	0.046466897	hsa-miR-561-5p	hsa-miR-140-3p
hsa_circRNA_104575	chr8	EPHX2	3.514241	0.047028967	hsa-miR-519e-5p	hsa-miR-519d-5p
hsa_circRNA_104852	chr9	RAD23B	3.3765236	0.006874968	hsa-miR-138-5p	hsa-miR-325
**Down-regulated circRNAs in older women**						
hsa_circRNA_101889	chr16	USP10	6.6744495	0.024280583	hsa-miR-670-3p	hsa-miR-103a-2-5p
hsa_circRNA_102273	chr17	B3GNTL1	5.4184314	0.030134376	hsa-miR-518a-5p	hsa-miR-527
hsa_circRNA_100332	chr1	PIP5K1A	4.2927661	0.034365002	hsa-miR-892a	hsa-miR-216a-3p
hsa_circRNA_103611	chr4	GPR125	3.2502319	0.047353991	hsa-miR-485-5p	hsa-miR-140-3p
hsa_circRNA_104379	chr7	GBAS	2.5310594	0.046807304	hsa-miR-185-3p	hsa-miR-96-5p
hsa_circRNA_102660	chr2	SLC30A6	2.437354	0.003288283	hsa-miR-141-5p	hsa-miR-449c-5p
hsa_circRNA_103612	chr4	SEPSECS	2.0950515	0.003948349	hsa-miR-649	hsa-miR-9-5p
hsa_circRNA_104457	chr7	PUS7	2.0716283	0.041742638	hsa-miR-599	hsa-miR-197-5p
hsa_circRNA_104755	chr9	UBAP2	2.0533773	0.012388518	hsa-miR-512-3p	hsa-miR-370-3p
hsa_circRNA_101852	chr16	SNTB2	2.05004	0.011694437	hsa-miR-424-5p	hsa-miR-15b-5p

Chrom, the chromosomal location of the circRNA; FC, fold change; *P*-value was calculated by t-test

### Stage 2: Identification and characterization of differentially expressed circRNAs in GCs during maternal aging

The top 10 up-regulated and top 4 down-regulated circRNAs ranked by fold change were selected for validation by qRT-PCR. As this was the first time to use these divergent primers for circRNAs quantification, it was necessary to test their specificity. Our melting curve analysis revealed that eight candidate transcripts showed a single peak (S1 Fig), including six up-regulated circRNAs (circRNA_103829, circRNA_103828, circRNA_103827, circRNA_ 100833, circRNA_104816 and circRNA_104852) and two down-regulated circRNAs (circRNA_101889 and circRNA_103611) in AA samples. Single-peak curves were not obtained from other six circRNAs, although three pairs of divergent primers were designed for each of them.

**Fig 2:**
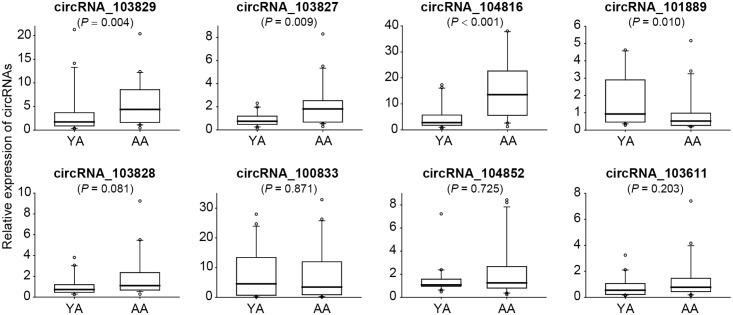
CircRNA expression variations in granulosa cells during maternal aging were validated by qRT-PCR. Comparison of candidate circRNAs (circRNA_103829, circRNA_10827, circRNA_104816, circRNA_101889, circRNA_103828, circRNA_100833, circRNA_104852, circRNA_103611) expression levels in granulosa cells from additional young (n = 20) and older (n = 20) women by qRT-PCR. YA, women with young age (≤ 30 years); AA, women with advanced age (≥ 38 years). *P* values were calculated by Mann-Whitney *U* test. Relative expressions were analyzed by 2^-ΔΔCt^ method which was normalized to GAPDH. For each box plotting, the central mark represents the median, the edges of the box represent the 25^th^ and 75^th^ percentiles, and the whiskers are the most extreme data points not considered outliers.

Then we compared the expression of these eight candidate circRNAs in GCs from additional 20 YA and 20 AA women by qRT-PCR. Among them, circRNA_103829, circRNA_103827 and circRNA_104816 were significantly up-regulated, while circRNA_101889 were remarkably down-regulated in AA women by Mann-Whitney *U* Test (*P* = 0.004, *P* = 0.009, *P* < 0.001, *P* = 0.010, respectively) (Fig 2). Likewise, Spearman’s correlation analysis showed that these four circRNAs levels were significantly associated with maternal age (r = 0.312, *P* = 0.050; r = 0.446, *P* = 0.004; r = 0.597, *P* < 0.001; r = -0.369, *P* = 0.019; respectively).

In addition to maternal age, GCs transcriptome profiles may be affected by gonadotropin treatment [37, 38]. Interestingly, we found that the expression of the four circRNAs was comparable in GCs from women who received agonist or antagonist protocols. Conversely, circRNA_101889 expression in GCs varied significantly according to the type of gonadotropin treatment (recombinant follicle-stimulating hormone (r-FSH) and/or highly purified human menopausal gonadotropin (HP-hMG) (*P* = 0.024) (S2 Fig). Besides, circRNA_103829 was significantly up-regulated in GCs from women who received high doses of gonadotropins (≥ 2500 IU/l) compared to those treated with doses < 2500 IU/l (*P* = 0.022) (S2 Fig).

After adjustment for gonadotropin treatment, only circRNA_103827 and circRNA_104816 levels were significantly and positively associated with maternal age in partial correlation analysis (partial r = 0.332, *P* = 0.045; partial r = 0.473, *P* = 0.003; respectively).

The unique circular characteristics of circRNA_103827 and circRNA_104816 were assessed by poly(A)- enrichment and RNase R digestion. As shown in S3 Fig, both circRNAs were abundant in poly(A)- fractions, barely detectable in poly(A)+ fractions, and resistant to 3’-5’ exoribonuclease RNase R digestion. In addition, their back-splice sequences were successfully detected in RNase R treated RNA (S3 Fig). These results strongly support that they are indeed circRNAs.

### Stage 3: Association of circRNA expression levels (circRNA_103827 and circRNA_104816) with diminished ovarian function

**Fig 3:**
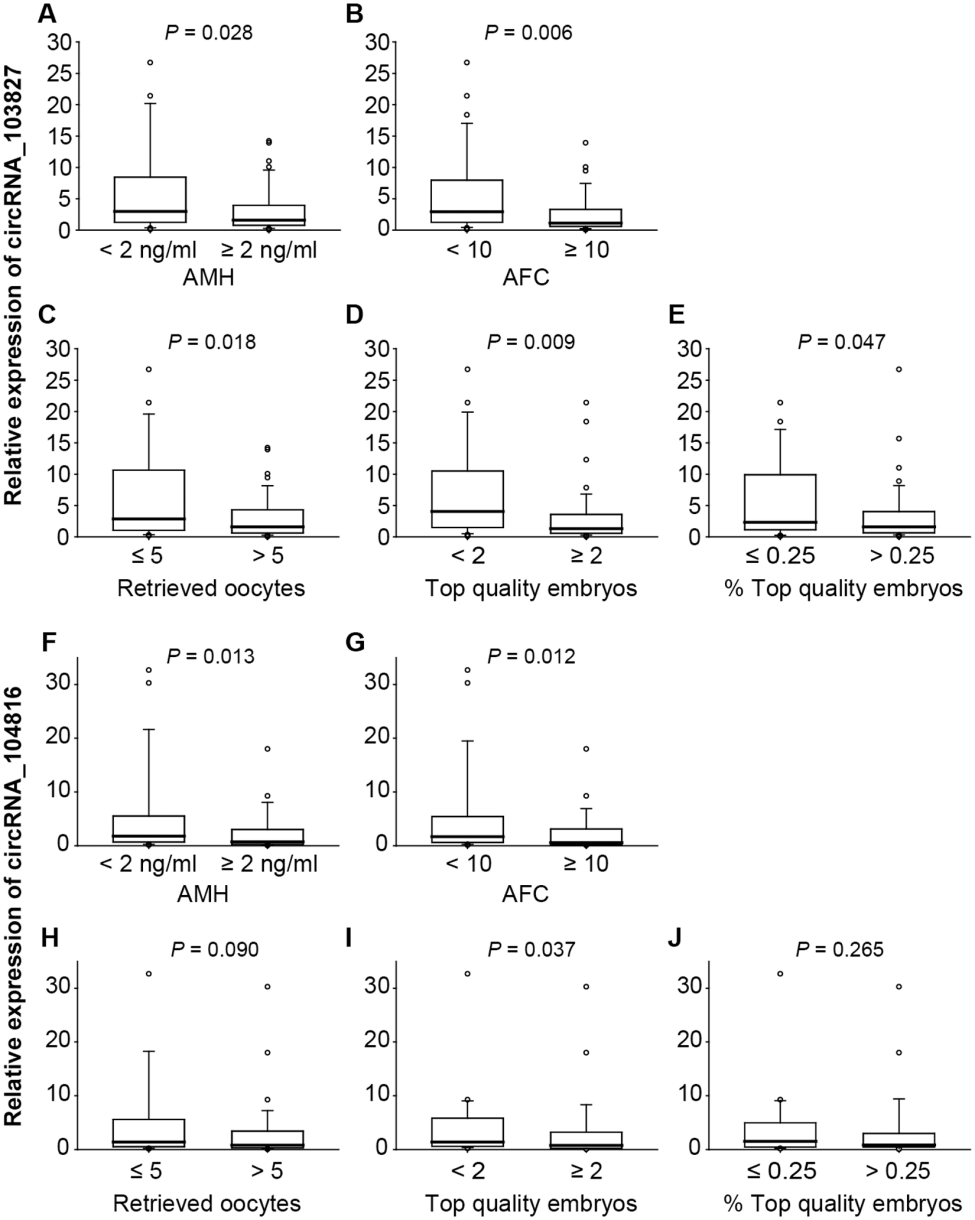
CircRNA_103827 and circRNA_104816 expression levels in granulosa cells according to ovarian reserve and embryo outcomes. (**A-E**) circRNA_103827 expression levels in granulosa cells according to (A) serum AMH levels, (B) AFC, (C) retrieved oocytes, (D) top quality embryos and (E) top quality embryo percentage. (**F-J**) circRNA_104816 expression levels in granulosa cells according to (F) serum AMH levels, (G) AFC, (H) retrieved oocytes, (I) top quality embryos and (J) top quality embryo percentage.

Considering the relationship between circRNAs and maternal age, we continued to investigate whether the variations of cirRNA_103827 and circRNA_104816 in GCs could reflect reduced ovarian reserve and oocyte quality in another cohort (80 women, aged from 22–48 years). Currently, ovarian reserve status is mainly evaluated by AMH and AFC, while oocyte quality could be preliminary assessed based on embryo development outcomes. As shown in Fig 3, circRNA_103827 and circRNA_104816 were significantly up-regulated in GCs from women with serum AMH concentration < 2ng/ml than in those with higher AMH levels (≥ 2ng/ml) (*P* = 0.028; *P* = 0.013; respectively). Spearman’s correlation analysis indicated a significant negative correlation of circRNA_103827 / circRNA_104816 levels and serum AMH concentration (r = -0.283, *P* = 0.011; r = -0.311, *P* = 0.005; respectively) (S5 Table). Moreover, circRNA_103827 and circRNA_104816 tended to be up-regulated in GCs from women with low AFC (< 10) than those from women with AFC ≥ 10 (*P* = 0.006; *P* = 0.012; respectively) (Fig 3 and Table 1). Indeed, both circRNA_103827 and circRNA_104816 expression levels were found to be significantly and negatively correlated with AFC (r = -0.305, *P* = 0.006; r = -0.314, *P* = 0.005; respectively) (S5 Table).

In addition to ovarian reserve parameters, GCs from women with a low number of retrieved oocytes (≤ 5) contained significant higher circRNA_103827 levels than those with higher number of oocytes (> 5) (*P* = 0.018) (Fig 3 and Table 2). Spearman’s correlation analysis also showed that circRNA_103827 levels in GCs were negatively correlated with the number of retrieved oocytes (r = -0.345, *P* = 0.002) (S6 Table), indicating the relationship between up-regulation of circRNA_103827 and poor ovarian response. On the other hand, circRNA_103827 and circRNA_104816 expressions did not vary significantly between different ovarian stimulation protocols (S7 Table).

For embryo outcomes at day 3 post-fertilization, we found that oocytes cohorts which gave rise to a small number of top quality embryos (< 2) were related to GCs with higher circRNA_103827 and circRNA_104816 expression levels than those achieving at least two top quality embryos (*P* = 0.009, *P* = 0.037, respectively) (Fig 3 and Table 2). The expression of circRNA_103827 and circRNA_104816 was also shown to be significantly and negatively correlated with the number of top quality embryos (r = -0.235, *P* = 0.036; r = -0.221, *P* = 0.049; respectively) (S6 Table). In addition, the ratio between number of top quality embryos and total number of mature oocytes was calculated to estimate global developmental competence of each oocyte cohort. CircRNA_103827 tended to be up-regulated in GCs of embryo cohorts with low proportion of top quality embryos (≤ 25%; decreased developmental competence) than those with top quality embryos > 25% (*P* = 0.047) (Fig 3 and Table 2). These data suggest that up-regulated circRNA_103827 and circRNA_104816 may be associated with adverse embryo outcomes.

### CircRNA_103827 and circRNA_104816 predictive values for pregnancy outcomes

**Fig 4:**
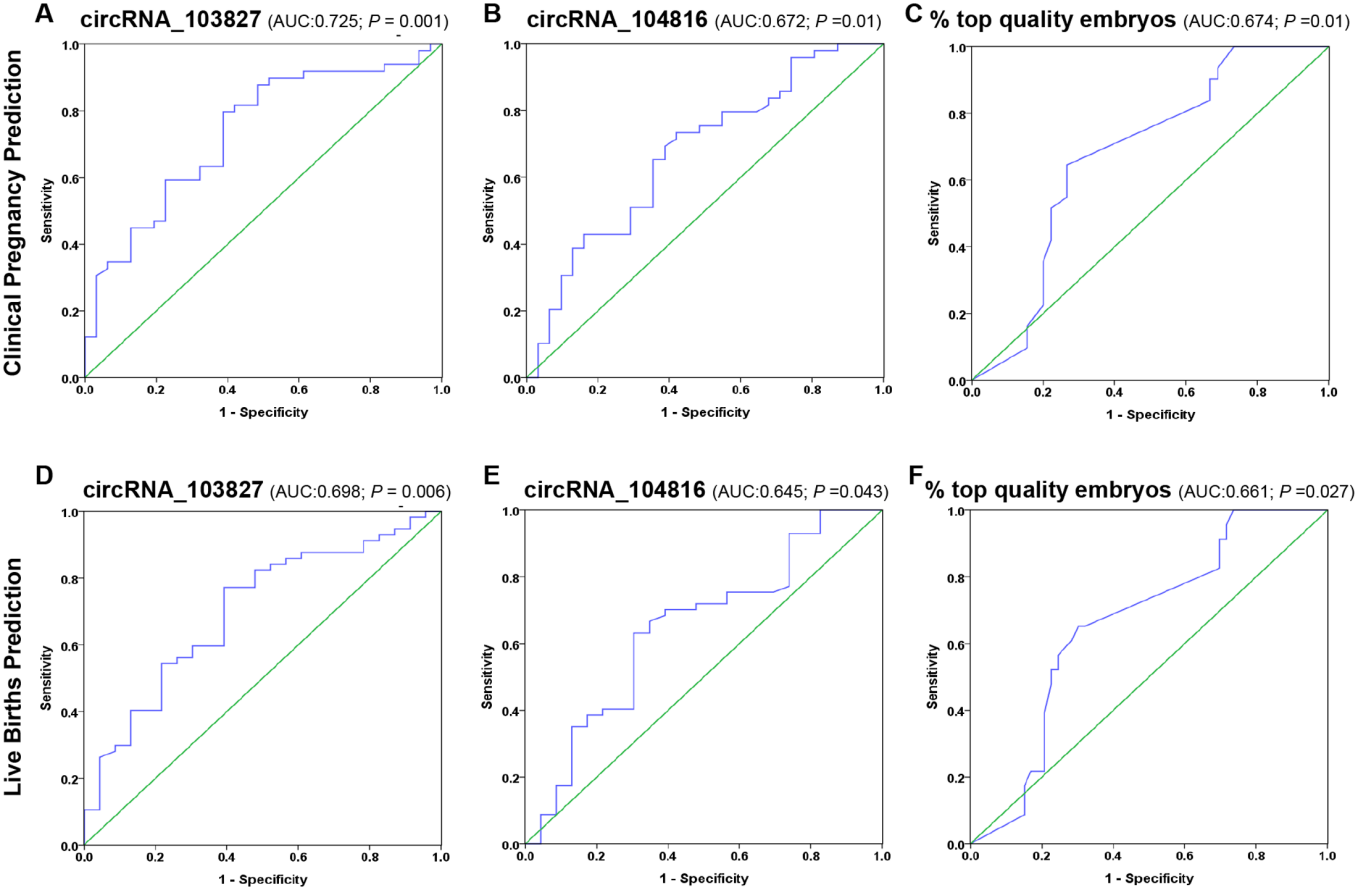
The predictive efficacy of circRNA_103827 and circRNA_104816 for pregnancy outcomes. ROC analysis to evaluate predictive performance of circRNA_103827, circRNA_104816 expressions in granulosa cells and top quality embryo proportion for (**A-C**) clinical pregnancy outcomes, as well as for (**D-F**) live births. AUC, area under the ROC curve.

Given that age-related decline in ovarian function is the most important factor for determining pregnancy success in women of advanced age [9], we further investigate whether the expression of cirRNA_103827 and circRNA_104816 in GCs could predict pregnancy outcomes in assisted reproduction. ROC curve analysis revealed the performance of circRNA_103827 for clinical pregnancy prediction was 0.725 [0.608–0.842] (*P* = 0.001), with 79.6% sensitivity and 61.3% specificity, and that of circRNA_104816 was 0.672 [0.550–0.795] (*P* = 0.01). Moreover, the predictive power of GCs circRNA_103827 expression for clinical pregnancy was higher than that of top quality embryo percentage (AUC = 0.674 [0.551–0.797]; *P* = 0.01) in our population (Fig 4A–4C).

Apart from clinical pregnancy, circRNA_103827 have predictive values for live births as well. ROC curve analysis indicated that the performance of circRNA_103827 for live birth prediction reached 0.698 [0.570–0.825] (*P* = 0.006), with 77.2% sensitivity and 60.9% specificity, and that of circRNA_104816 was 0.645 [0.507–0.783] (*P* = 0.043). In our population, the power of circRNA_103827 expression for live birth prediction was higher than that of top quality embryo percentage (AUC = 0.661 [0.533–0.789], *P* = 0.027) (Fig 4D–4F).

### Prediction and annotation of circRNA_103827 and circRNA_104816 targeted miRNA-gene network

**Fig 5:**
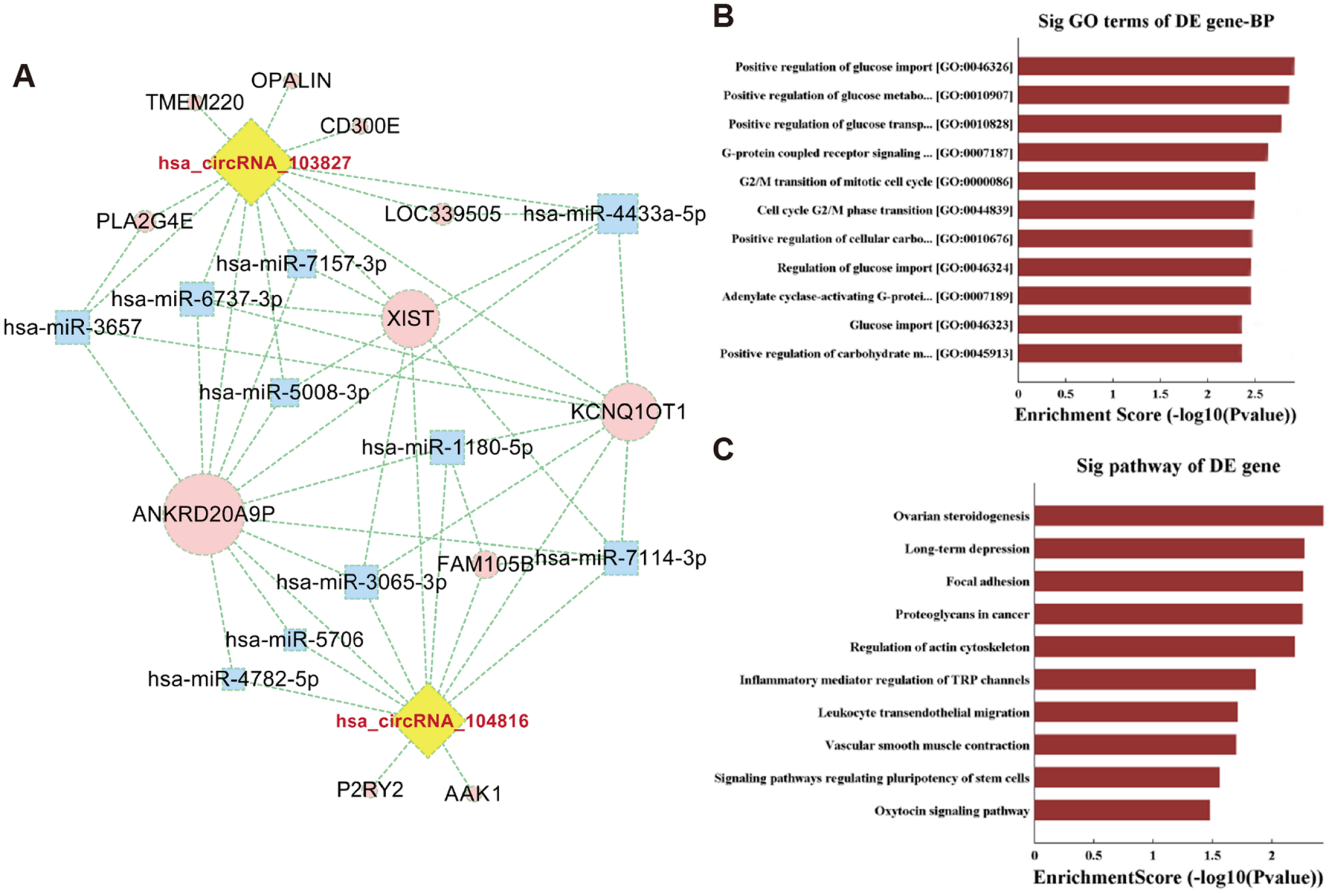
Potential biological functions of circRNA_103827 and circRNA_104816. **(A)** CircRNA_103827/circRNA_104816 targeted “Top 5” miRNA-gene network was portrayed based on sequence-pairing prediction. A pink round node represents a gene, a blue square represents miRNA, and a yellow diamond represents a circRNA. Overlapping genes of both circRNAs in this interactive network were *ANKRD20A9P*, *XIST* and *KCNQ1OT1*. **(B)** GO and **(C)** KEGG pathway analysis of circRNA_103827 and circRNA_104816 predicted target genes. The top 10 significantly enriched activities and their scores (negative logarithm of *P* value) were listed in the X-axis and the Y-axis, respectively.

To explore biological function of circRNA_103827 and circRNA_104816, we used bioinformatics to predict their potential targets and interactive networks. Given that circRNAs were reported to function as miRNA sponges [23], we assumed that circRNA_103827 and circRNA_104816 may regulate gene expression by a similar mechanism. The molecular interaction of circRNA_103827 and circRNA_104816 with their five highest ranking candidate miRNA targets (Top 5) based on mirSVR scores were depicted in S4 Fig. Since the complete competing endogenous RNA (ceRNA) relationship could not be presented clearly in a map, we constructed an interactive network with two circRNAs and their respective relevant top 5 miRNAs and top 6 genes. Interestingly, this network revealed that circRNA_103827 and circRNA_104816 may regulate the same target genes, including *ANKRD20A9P*, *KCNQ1OT1* and *XIST* (Fig 5A and S5 Fig).

To gain further insights into functions of circRNA_103827 and circRNA_104816, GO and KEGG pathway analysis were utilized based on their predicted targets. CircRNA_103827 and circRNA_104816 showed a strong relationship with biological processes of glucose import, glucose metabolic process, glucose transport, G-protein coupled receptor signaling pathway, and G2/M transition of mitotic cell cycle **(**Fig 5B). Pathway analysis revealed that circRNA_103827 and circRNA_104816 were predicted to be involved in ovarian steroidogenesis **(**Fig 5C).

## Discussion

Ovarian aging, termed as decline of ovarian function with increasing age, is considered as the trigger of female aging and subfertility, and it is associated with many age-related diseases such as osteoporosis and cardiac vascular diseases [5, 39]. Prolonged human lifespan and postponed childbearing prompt researchers to investigate mechanisms underlying ovarian aging. Extensive studies have focused on characterizing mRNA and miRNAs profiles in human GCs according to maternal age [10, 15], and expanded our knowledge of molecular processes occurring in follicular cells. However, the application of new sequencing technologies provides opportunities to study other novel molecules with promising prospects.

In this study, we introduced circRNAs to shed new light on ovarian aging mechanisms. There are three important properties for circRNAs: first, they are highly conserved and tissue-specific sequences; second, they can tolerate exonuclease degradation, and exhibit high degree of stability in mammalian cells; third, they have unique ceRNA characteristics [17, 23, 40]. Compared with other linear RNAs, these properties provide circRNAs with the potential to be used as ideal biomarkers and therapeutic targets. Therefore, it is worth exploring circRNAs in human reproduction.

Our study is the first to profile circRNA expression patterns of human GCs according to female age, and uncovered a general trend of circRNA up-regulation in GCs during aging. This age-accumulation trend of circRNA was previously reported in mouse brain tissues [24]. Specifically, circRNA_103827 and circRNA_104816 in GCs were found to be up-regulated in older women. Moreover, the expression of the two circRNAs were negatively correlated with serum AMH levels and AFC, which have been demonstrated to be excellent biomarkers of ovarian reserve [41, 42]. This relationship indicates the important role of circRNAs in ovarian aging. Considering the high dispersion of circRNA_103827 and circRNA_104816 expressions in populations, they may not be suitable for use as predictors of ovarian function alone, but serve as complementary indicators.

Oocyte quality, which can be influenced and reflected by follicular micro-environment, has been well recognized as a key limiting factor in female fertility [9]. Many investigations have tried to use proteins, mRNA and miRNAs as surrogate biomarkers for predicting oocyte developmental competence and pregnancy outcomes after ART [10, 14, 43–45]. Despite some progress, the universally applicable and non-invasive test for the determinant of pregnancy outcomes has not yet been established. For example, the performance of serum AMH for pregnancy prediction was 0.634 (95% CI, 0.618–0.650), with 44.0% sensitivity and 66.5% specificity [46]. In addition, the predictive power of FF miR-29a levels for clinical pregnancy was 0.68 (95% CI, 0.55–0.79) with a sensitivity of 83.3%, but a low specificity (53.5%) [47]. In our study, circRNA_103827 and circRNA_104816 expressions in GCs were found to be negatively associated with embryonic development. The predictive performance of circRNA_103827 expression in GCs for clinical pregnancy was 0.725, with 79.6% sensitivity and 61.3% specificity, and that of circRNA_104816 was 0.672, with 74.1% sensitivity and 57.2% specificity. Thus, these indicators have their own advantages in terms of sensitivity and specificity. Combination of proteins, mRNAs or microRNAs in previous studies and circRNAs in this study may be beneficiary for clinical evaluation of ovarian function and pregnancy outcomes. Our study adds new information and promising biomarkers to the field. Given the effect of population heterogeneity, the results need to be validated in larger number size and other populations.

For potential biological functions, circRNA_103827/circRNA_104816-miRNA-mRNA networks suggest that both circRNAs may regulate common target genes, *ANKRD20A9P*, *KCNQ1OT1* and *XIST*. *ANKRD20A9P* has been identified as a novel loci associated with longevity in Han Chinese GWAS [48]. *XIST* (X-inactive specific transcript) plays critical roles in the initiation of imprinted X-chromosome inactivation. In females, lack of *XIST* leads to genome-wide transcriptional misregulation in early blastocysts and post-implantation lethality [49]. *KCNQ1OT1* is a paternally expressed allele involved in the transcriptional silencing by regulating histone methylation, and its misregulation could lead to disastrous physical and genetic abnormalities [50]. Epigenetic dysregulation has been identified as an important mechanism underlying ovarian aging and abnormal embryonic development [51, 52]. Accordingly, the critical roles of *XIST* and *KCNQ1OT1* in epigenetics support our hypothesis that age-related up-regulation of circRNA_103827 and circRNA_104816 may be involved in ovarian aging.

Investigations of GCs could contribute to understanding compromised follicular micro-environment in the aging ovary, and provide insight into the mechanisms underlying reduced oocyte quality. Based on previous studies, GCs from women aged > 38 years showed decreased proliferation, reduced steroidogenic potential, increased apoptotic changes, abnormal glucose metabolism and more damaged mitochondria compared to younger women [7, 8]. Proteomic analysis revealed that genes involved in steroid hormone synthesis, oxidative phosphorylation and post-transcriptional splicing processes were down-regulated with advancing female age [16]. Our in silico prediction suggests that circRNA_103827 and circRNA_104816 were potentially involved in glucose metabolism, mitotic cell cycle, and ovarian steroidogenesis. Therefore, age-related up-regulation of circRNA_103827 and circRNA_104816 may be responsible for follicular micro-environment deterioration with consequences for poor oocyte and embryo quality. Next, we will explore corresponding circRNA functions using overexpression and knockdown experiments.

Despite these novel and promising findings in this study, there were still some weaknesses we recognized. Firstly, one limitation is the small sample size in microarray analysis which may decrease statistical power. Considering this limitation, we expanded the sample size to 20 patients in each arm for subsequent validation to obtain credible results. Another limitation is heterogeneity in the study population. However, we controlled other confounding factors such as PCOS and endometriosis to minimize the differences, and adjusted ovarian stimulation protocols by statistical analysis. Thirdly, the physiological significance of the circRNAs in reproduction is only predicted. The novel identified circRNAs associated with ovarian aging and the prediction of their potential functions laid a solid foundation for further investigation.

In conclusion, our study was the first to characterize global circRNA expression patterns in human GCs based on female age, and demonstrated the significant difference between young and older women. Elevated expression of circRNA_103827 and circRNA_104816 were closely related to declining ovarian reserve and adverse reproductive outcomes. Intriguingly, circRNA_103827 has predictive performance for pregnancy outcomes after ART cycles. This study sheds new light on altered follicular micro-environment in aging ovary and adds novel transcripts associated with reproductive outcomes. Deciphering molecular mechanisms and biological functions of circRNAs would improve personalized ART strategies and identify new biomarkers and therapeutic targets in infertility female with advanced age.

## Supporting information

S1 Fig Melting curves of qRT-PCR products of these candidate circRNAs. (TIF)

S2 Fig Effect of gonadotropins treatment on circRNA expressions in human granulosa cells. (**A**) CircRNA_101889 expression levels according to the type of gonadotropins. (**B**) CircRNA_103829 expression levels according to total dose of gonadotropins. r-FSH, recombinant follicle-stimulating hormone; HP-hMG, highly purified human menopausal gonadotropin.
(TIF)

S3 Fig Characterization of circRNA_103827 and circRNA_104816 in human granulosa cells. **(A)** Expression of circRNA_103827 and circRNA_104816 in poly(A)+/− RNA were detected by RT-PCR using divergent primers and agarose gel electrophoresis. GAPDH was used as polyadenylated positive control. (**B**) The expression of circRNA_103827 and circRNA_104816 in granulosa cells RNA with (+) or without (−) RNase R digestion. GAPDH was used as RNase-sensitive control. (**C**) Amplification of specific back-splice sequence of circRNA_103827 and circRNA_104816 in granulosa cells by RT-PCR and Sanger sequencing. “c103829” represented as “circRNA_103929”, and so forth.
(TIF)

S4 Fig Detailed molecular interaction between circRNAs and their miRNA targets. The Top5 candidate miRNA targets of (**A**) circRNA_103827 and (**B**) circRNA_104816 were respectively predicted by miSVR. The molecular interaction of circRNA with its miRNA targets was based on complementary base pairing principle.
(TIF)

S5 Fig The predicted interactive circRNA-miRNA-mRNA network between circRNA_103827 and circRNA_104816. “Top 4” miRNA of the largest interaction with other nodes in both ceRNAs maps, including miR-98-5p, miR-3187-5p, miR-4458 and miR-4500 were shown in this network.
(TIF)

S1 Table Clinical characteristics and assisted reproductive technology outcomes of the patients used for microarray analysis in stage one. YA, young age; AA, advanced age; BMI, body mass index; FSH, follicle-stimulating hormone; LH, luteinizing hormone; E2,17β-estradiol; T, testosterone; PRL, prolactin; AMH, anti-Müllerian hormone; AFC, antral follicle count; IVF, *in vitro* fertilization; ICSI, intracytoplasmic sperm injection; Gn, gonadotropin; OPU, oocyte pick-up; COCs, cumulus oocyte complexes; MII, oocyte blocked in meiotic metaphase II; 2PN, 2-pronuclear fertilization; Good quality embryos, embryos with 7–8 regular blastomeres and less than 10% fragments on day 3.
(DOCX)

S2 Table Clinical characteristics of women used for candidate circRNAs validation in stage two. YA, young age; AA, advanced age; E2,17β-estradiol; PRL, prolactin; T, testosterone; AMH, anti-Müllerian hormone; AFC, antral follicle count; IVF, *in vitro* fertilization; ICSI, Intracytoplasmic sperm injection. Gn, gonadotropin; P4, progesterone; NS, no significance (*P* ≥ 0.05). Numerical values are in the form of Mean ± SD. ^a^ Data were analyzed by two-tailed *t* test; ^b^ Chi-square test. * *P* < 0.05; ** *P* < 0.01.
(DOCX)

S3 Table The detailed information of primers used in PCR. (DOCX)

S4 Table The significantly differentially expressed circRNAs between YA and AA samples. FC, fold change; MRE, miRNA response elements.
(DOCX)

S5 Table The correlation coefficients (r) of circRNAs and clinical variables (n = 80) were analyzed by Spearman. (DOCX)

S6 Table The correlation coefficients (r) of circRNAs and ART outcomes (n = 80) were analyzed by Spearman. (DOCX)

S7 Table CircRNA_103827 and circRNA_104816 expression levels in granulosa cells relative to ovarian stimulation protocols. r-FSH, recombinant follicle-stimulating hormone; HP-hMG, highly purified human menopausal gonadotropin; E2,17β-estradiol. * *P* < 0.05; ** *P* < 0.01.
(DOCX)

S1 File STROBE statement—Checklist of items that should be included in reports of case-control studies. (DOC)
