# Supplementary figures and images for: Circular RNA expression profiling of human granulosa cells during maternal aging reveals novel transcripts associated with assisted reproductive technology outcomes
Source: PLoS One. 2017 Jun 23;12(6):e0177888. doi: 10.1371/journal.pone.0177888 (PMC5482436; doi:10.1371/journal.pone.0177888)

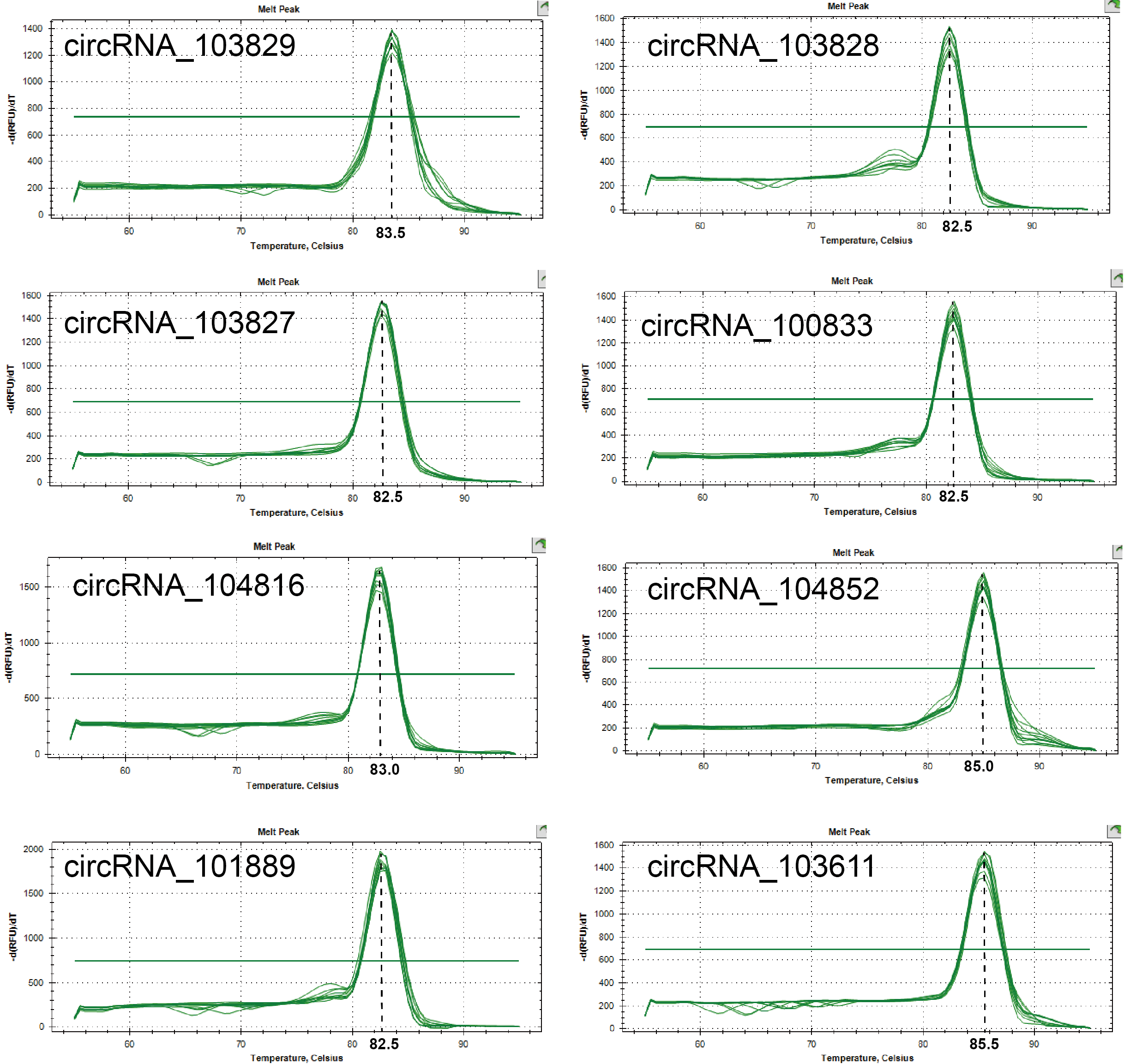

Supplement: S1 Fig — (TIF) [file pone.0177888.s001.tif]

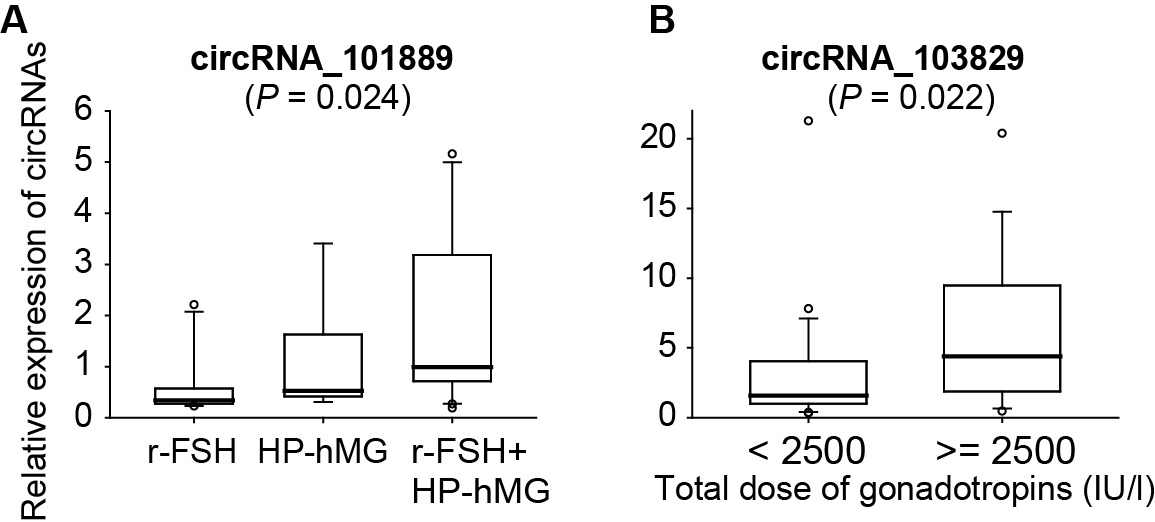

Supplement: S2 Fig — (A) CircRNA_101889 expression levels according to the type of gonadotropins. (B) CircRNA_103829 expression levels according to total dose of gonadotropins. r-FSH, recombinant follicle-stimulating hormone; HP-hMG, highly purified human menopausal gonadotropin. (TIF) [file pone.0177888.s002.tif]

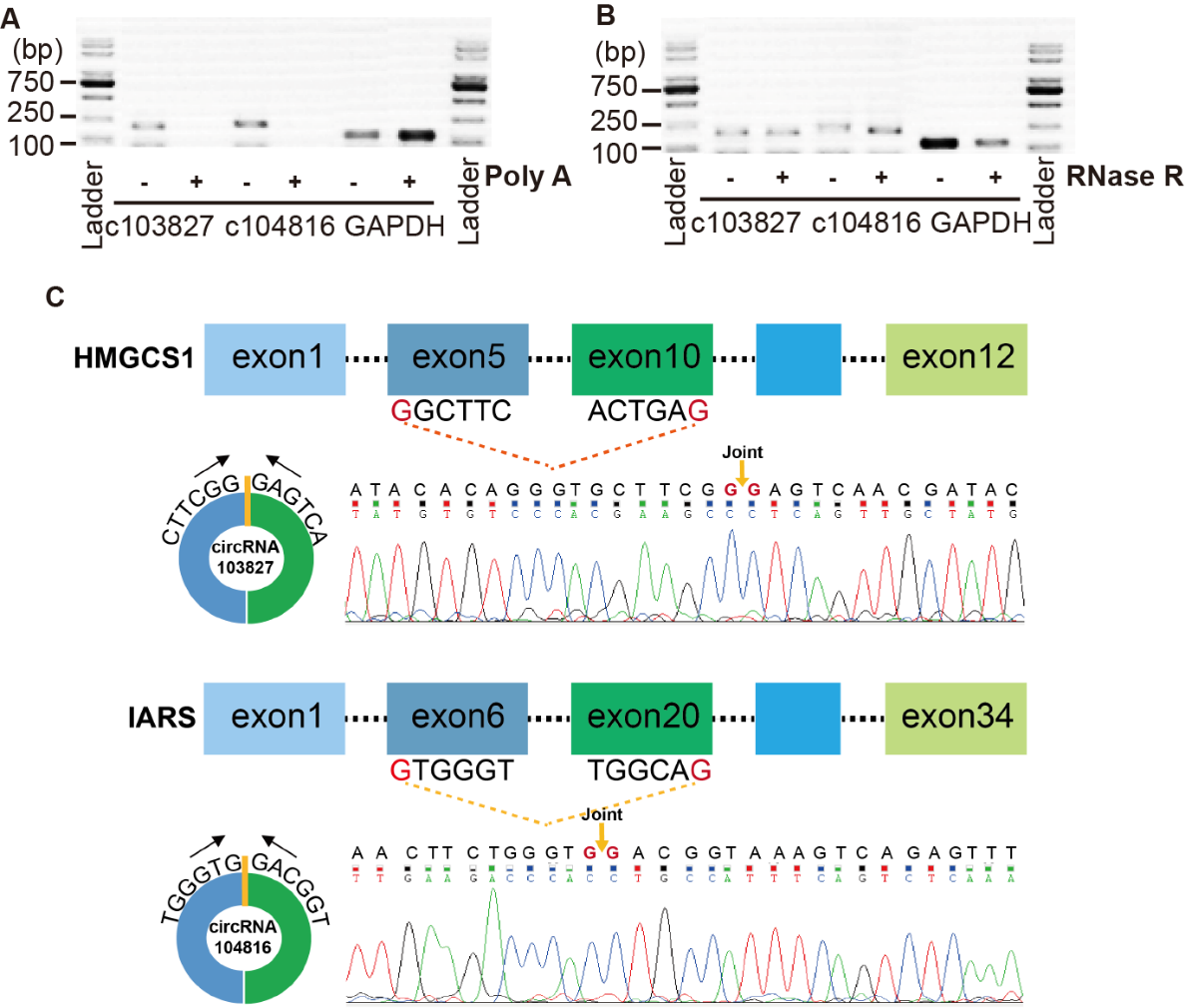

Supplement: S3 Fig — (A) Expression of circRNA_103827 and circRNA_104816 in poly(A)+/− RNA were detected by RT-PCR using divergent primers and agarose gel electrophoresis. GAPDH was used as polyadenylated positive control. (B) The expression of circRNA_103827 and circRNA_104816 in granulosa cells RNA with (+) or without (−) RNase R digestion. GAPDH was used as RNase-sensitive control. (C) Amplification of specific back-splice sequence of circRNA_103827 and circRNA_104816 in granulosa cells by RT-PCR and Sanger sequencing. “c103829” represented as “circRNA_103929”, and so forth. (TIF) [file pone.0177888.s003.tif]

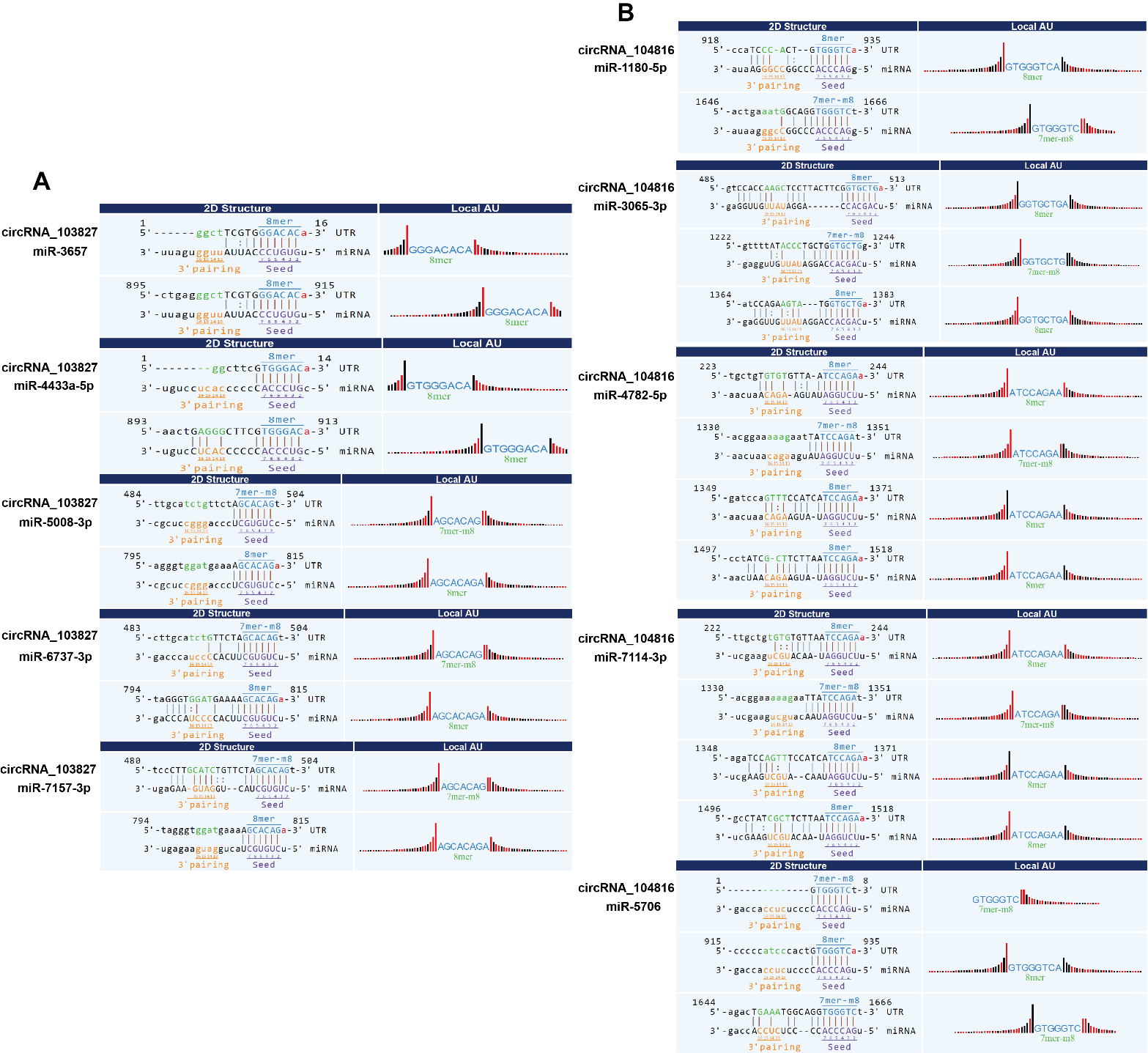

Supplement: S4 Fig — The Top5 candidate miRNA targets of (A) circRNA_103827 and (B) circRNA_104816 were respectively predicted by miSVR. The molecular interaction of circRNA with its miRNA targets was based on complementary base pairing principle. (TIF) [file pone.0177888.s004.tif]

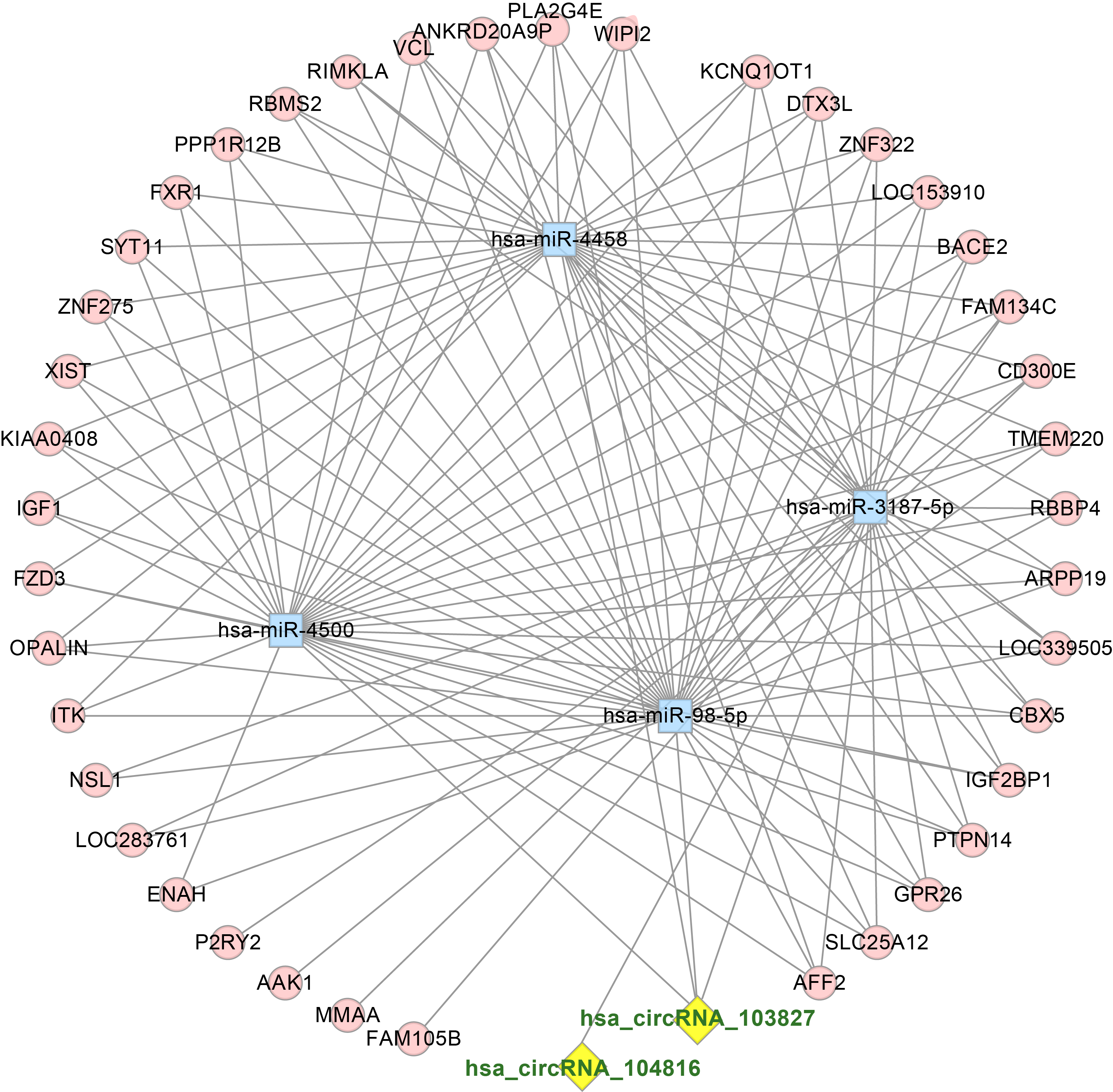

Supplement: S5 Fig — “Top 4” miRNA of the largest interaction with other nodes in both ceRNAs maps, including miR-98-5p, miR-3187-5p, miR-4458 and miR-4500 were shown in this network. (TIF) [file pone.0177888.s005.tif]
