# Supplementary material for: Circular RNA expression profiling of human granulosa cells during maternal aging reveals novel transcripts associated with assisted reproductive technology outcomes
Source: PLoS One. 2017 Jun 23;12(6):e0177888. doi: 10.1371/journal.pone.0177888 (PMC5482436; doi:10.1371/journal.pone.0177888)
Supplement: S1 Table — YA, young age; AA, advanced age; BMI, body mass index; FSH, follicle-stimulating hormone; LH, luteinizing hormone; E2,17β-estradiol; T, testosterone; PRL, prolactin; AMH, anti-Müllerian hormone; AFC, antral follicle count; IVF, in vitro fertilization; ICSI, intracytoplasmic sperm injection; Gn, gonadotropin; OPU, oocyte pick-up; COCs, cumulus oocyte complexes; MII, oocyte blocked in meiotic metaphase II; 2PN, 2-pronuclear fertilization; Good quality embryos, embryos with 7–8 regular blastomeres and less than 10% fragments on day 3. (DOCX) [file pone.0177888.s006.docx]

| **S1 Table. Clinical characteristics and assisted reproductive technology outcomes of the patients used for microarray analysis in stage one.** | | | | | | | | | |
| --- | --- | --- | --- | --- | --- | --- | --- | --- | --- |
| **Variable** | **YA (≤ 30 years)** | | | | **AA (≥ 38 years)** | | | | ***P* value** |
| Age (years) | | 24 | 24 | 23 | | 40 | 43 | 41 | **<0.001** ^a^ |
| BMI (kg/m^2^) | | 26.3 | 23.2 | 21.8 | | 24.2 | 21.2 | 22.8 | 0.551 ^a^ |
| Basal FSH (IU/L) | | 5.29 | 6.13 | 4.89 | | 9.41 | 8.57 | 8.16 | **0.003** ^a^ |
| Basal LH (IU/L) | | 1.27 | 2.42 | 2.79 | | 3.19 | 9.22 | 1.69 | 0.340 ^a^ |
| Basal E2 (pg/ml) | | 28 | 49.93 | 31.12 | | 38.53 | 22.1 | 43.08 | 0.858 ^a^ |
| Basal T (ng/dl) | | 32.28 | 43.81 | 53.19 | | 36.7 | 50.06 | 42.15 | 0.987 ^a^ |
| Basal PRL (ng/ml) | | 12.78 | 11.46 | 9.6 | | 12.51 | 19.96 | 8.86 | 0.503 ^a^ |
| AMH (ng/ml) | | 8.21 | 4.39 | 9.26 | | 1.7 | 0.49 | 2.86 | **0.026** ^a^ |
| Inhibin B (pg/ml) | | 89.42 | 183.91 | 163.64 | | 48.58 | 57.39 | 86.42 | 0.058 ^a^ |
| AFC (n) | | 21 | 20 | 18 | | 7 | 6 | 5 | **<0.001** ^a^ |
| IVF / ICSI | | ICSI | ICSI | IVF | | IVF | IVF | ICSI | 0.456 ^b^ |
| Infertility aetiology | | Male | Male | Female (Tubal) | | Female (AA) | Female (AA) | Mixed | 0.099 ^b^ |
| Stimulation days | | 9 | 12 | 10 | | 7 | 6 | 9 | 0.074 ^a^ |
| Gn administered (IU) | | 1800 | 2287 | 1012 | | 2050 | 3600 | 1875 | 0.289 ^a^ |
| E2 level of OPU (pg/ml) | | 3871 | 2478 | 6760 | | 989 | 615.32 | 624 | **0.046** ^a^ |
| P4 of OPU (ng/ml) | | 0.9 | 1.28 | 0.81 | | 1.25 | 0.96 | 0.46 | 0.715 ^a^ |
| Retrieved COCs (n) | | 14 | 17 | 16 | | 2 | 1 | 3 | **<0.001** ^a^ |
| MII oocytes (n) | | 12 | 14 | 11 | | 2 | 1 | 2 | **<0.001** ^a^ |
| 2PN (n) | | 10 | 11 | 10 | | 1 | 1 | 2 | **<0.001** ^a^ |
| Top quality embryos (n) | | 6 | 7 | 9 | | 0 | 0 | 1 | **0.002** ^a^ |
| Blastocysts (n) | | 4 | 9 | 8 | | — | — | 0 | **0.010** ^a^ |
| Top quality blastocysts (n) | | 3 | 5 | 6 | | — | — | 0 | **0.006** ^a^ |
| Clinical pregnancy | | Yes | Yes | Yes | | — | — | No | **0.025** ^b^ |
| Live birth | | Singleton | Twin | Singleton | | — | — | No | **0.025** ^b^ |
| YA, young age; AA, advanced age; BMI, body mass index; FSH, follicle-stimulating hormone; LH, luteinizing hormone; E2,17β-estradiol; T, [testosterone](javascript:void(0);); PRL, prolactin; AMH, anti-Müllerian hormone; AFC, antral follicle count; IVF, *in vitro* fertilization; ICSI, intracytoplasmic sperm injection; Gn, gonadotropin; OPU, oocyte pick-up; COCs, cumulus oocyte complexes; MII, oocyte blocked in meiotic metaphase II; 2PN, 2-pronuclear fertilization; Top quality embryos, embryos with 7–8 regular blastomeres and less than 10% fragments on day 3.  ^a^ Data were analyzed by two-tailed *t* test; ^b^ Mann-Whitney *U* test. | | | | | | | | | |
