# Supplementary material for: Circular RNA expression profiling of human granulosa cells during maternal aging reveals novel transcripts associated with assisted reproductive technology outcomes
Source: PLoS One. 2017 Jun 23;12(6):e0177888. doi: 10.1371/journal.pone.0177888 (PMC5482436; doi:10.1371/journal.pone.0177888)
Supplement: S2 Table — YA, young age; AA, advanced age; E2,17β-estradiol; PRL, prolactin; T, testosterone; AMH, anti-Müllerian hormone; AFC, antral follicle count; IVF, in vitro fertilization; ICSI, Intracytoplasmic sperm injection. Gn, gonadotropin; P4, progesterone; NS, no significance (P ≥ 0.05). Numerical values are in the form of Mean ± SD. a Data were analyzed by two-tailed t test; b Chi-square test. * P < 0.05; ** P < 0.01. (DOCX) [file pone.0177888.s007.docx]

| **S2 Table. Clinical characteristics of women used for candidate circRNAs validation in stage two.** | | | | |
| --- | --- | --- | --- | --- |
| **Variables** | | **YA (n=20)** | **AA (n=20)** | ***P* value** |
| Maternal age (years) | | 26.80 ± 2.64 | 40.70 ± 2.27 | ***P* < 0.001**** ^a^ |
| BMI (kg/m^2^) | | 21.40 ± 2.72 | 22.38 ± 2.81 | *P* = 0.27 ^a^ |
| Baseline evaluation | Basal FSH (IU/L) | 6.97 ± 1.68 | 10.50 ± 5.54 | ***P =* 0.012*** ^a^ |
|  | Basal LH (IU/L) | 4.18 ± 2.03 | 4.08 ± 2.30 | *P* = 0.106 ^a^; NS |
|  | Basal E2 (pg/ml) | 47.65 ± 21.51 | 42.58 ± 11.91 | *P* = 0.362 ^a^; NS |
|  | Basal PRL (ng/ml) | 16.51 ± 9.37 | 15.16 ± 5.95 | *P* = 0.591 ^a^; NS |
|  | Basal T (pg/ml) | 40.99 ± 15.54 | 34.01 ± 11.08 | *P* = 0.085 ^a^; NS |
|  | AMH (ng/ml) | 4.96 ± 2.40 | 1.44 ± 0.89 | ***P* < 0.001**** ^a^ |
|  | Inhibin B (ng/ml) | 119.18 ± 46.39 | 85.98 ± 51.12 | ***P* = 0.038*** ^a^ |
|  | AFC (n) | 16.10 ± 5.19 | 5.90 ± 2.86 | ***P* < 0.001**** ^a^ |
| Infertility aetiology | Male factor (n, %) | 9 (45) | 2 (10) | *P* = 0.079 ^b^; NS |
|  | Female factor (n, %) | 5 (25) | 11 (55) |  |
|  | Mixed (n, %) | 5 (25) | 6 (30) |  |
|  | Unexplained (n, %) | 1 (5) | 1 (5) |  |
| Diagnosis | Primary infertility (n, %) | 13 (65) | 9 (45) | *P* = 0.341 ^b^; NS |
|  | Secondary infertility (n, %) | 7 (35) | 11 (55) |  |
| Treatment | IVF (n, %) | 9 (45) | 15 (75) | *P* = 0.105 ^b^; NS |
|  | ICSI (n, %) | 11 (55) | 5 (25) |  |
| Stimulation | Agonist protocol (n, %) | 12 (60) | 8 (40) | *P* = 0.430 ^b^; NS |
|  | Antagonist protocol (n, %) | 7 (35) | 11 (55) |  |
|  | Days of stimulation | 10.4 ± 1.35 | 9.75 ± 3.32 | *P* = 0.425 ^a^; NS |
|  | Total Gn dose（IU） | 2021.70 ± 576.66 | 2365.60 ± 991.59 | *P* = 0.188 ^a^; NS |
|  | The type of Gn | | | |
|  | r-FSH (n, %) | 5 (25) | 5 (25) | *P* = 0.067 ^b^; NS |
|  | HP-hMG (n, %) | 2 (10) | 8 (40) |  |
|  | r-FSH + HP-hMG (n, %) | 13 (65) | 7 (35) |  |
| Hormonal levels at oocyte pick up | Peak E2 level (pg/ml) | 3807.65 ± 1683.52 | 1094.38 ± 989.47 | ***P* < 0.001**** ^a^ |
|  | P4 level (ng/ml) | 1.09 ± 0.35 | 0.66 ± 0.42 | ***P* = 0.001**** ^a^ |
| YA, young age; AA, advanced age; E2,17β-estradiol; PRL, prolactin; T, [testosterone](javascript:void(0);); AMH, anti-Müllerian hormone; AFC, antral follicle count; IVF, *in vitro* fertilization; ICSI, Intracytoplasmic sperm injection. Gn, gonadotropin; P4, [progesterone](javascript:void(0);); NS, no significance (*P* ≥ 0.05).  Numerical values are in the form of Mean ± SD.  ^a^ Data were analyzed by two-tailed *t* test; ^b^ Chi-square test.  * *P* < 0.05; ** *P* < 0.01. | | | | |
