# Supplementary material for: Circular RNA expression profiling of human granulosa cells during maternal aging reveals novel transcripts associated with assisted reproductive technology outcomes
Source: PLoS One. 2017 Jun 23;12(6):e0177888. doi: 10.1371/journal.pone.0177888 (PMC5482436; doi:10.1371/journal.pone.0177888)
Supplement: S3 Table — (DOCX) [file pone.0177888.s008.docx]

| **S3 Table. The detailed information of primers used in PCR.** | | | | |  |
| --- | --- | --- | --- | --- | --- |
| **Primer name** | **Sequence (5'-3')** | | **Product size (bp)** | | |
|  | **Forward** | **Reverse** |  |  |  |
| **Primers used for candidate circRNAs' characterization and validation.** | | | | | |
| circRNA_103829 | GCACAGTACTCACCTCAGCA | GCGGTCTAATGCACTGAGGT | | 231 | |
| circRNA_103828 | GGCGTCCCACTCCAAATGAT | ACATCTTTTGGCCAGCAAGC | | 130 | |
| circRNA_103827 | CAGAAGAACTTACGCTCGGC | ATGCACTGAGGTAGCACTGT | | 199 | |
| circRNA_100833 | AATCATCGCCACTTCCAGCA | AGTTCACCAATCAGCAGGGG | | 179 | |
| circRNA_104816 | GAACGTTCTGAGGCTCCAGA | AGTGGAGTGTTACATGCCGT | | 230 | |
| circRNA_104852 | CCACACCTGCATCCATCACT | CAGTTGTGGTGGTGGAGGAA | | 307 | |
| circRNA_101889 | AGAGCTTGGTGGCAAGAGAA | ACCAATGCCTGCAGTGTAGC | | 165 | |
| circRNA_103611 | CCTCCTGCATCCTGTGGTTT | CTGGACATGACAGCCCCAAT | | 271 | |
| GAPDH | CCAGCAAGAGCACAAGAGGA | ACATGGCAACTGTGAGGAGG | | 104 | |
| **Primers used for amplification of back-splice sequences in RT-PCR.** | | | | | |
| circRNA_103827 | GCACCAGATGTCTTCGCTGA | GCAGCGGTCTAATGCACTGA | | 341 | |
| circRNA_104816 | AGCCCCAACATTACAGACCG | CCAGGGAGTGGTTGTCCAAG | | 281 | |
