# Supplementary material for: Circular RNA expression profiling of human granulosa cells during maternal aging reveals novel transcripts associated with assisted reproductive technology outcomes
Source: PLoS One. 2017 Jun 23;12(6):e0177888. doi: 10.1371/journal.pone.0177888 (PMC5482436; doi:10.1371/journal.pone.0177888)
Supplement: S4 Table — FC, fold change; MRE, miRNA response elements. (DOCX) [file pone.0177888.s009.docx]

| **S4 Table. The significantly differentially expressed circRNAs between YA and AA samples.** | | | | | | | |
| --- | --- | --- | --- | --- | --- | --- | --- |
| **Rank** | **circRNA** | **Alias** | **Gene Symbol** | **FC** | ***P*-value** | **MRE** | |
| **Up-regulated Differentiation (AA VS YA)** | | | | | | | |
| 1 | hsa_circRNA_102476 | hsa_circ_0007396 | MYO9B | 5.4408000 | 0.0220997 | hsa-miR-767-3p | hsa-miR-877-3p |
| 2 | hsa_circRNA_103829 | hsa_circ_0072387 | HMGCS1 | 5.2789000 | 0.0095795 | hsa-miR-625-3p | hsa-miR-129-5p |
| 3 | hsa_circRNA_103828 | hsa_circ_0072386 | HMGCS1 | 4.8616000 | 0.0011755 | hsa-miR-411-5p | hsa-miR-625-3p |
| 4 | hsa_circRNA_103827 | hsa_circ_0008621 | HMGCS1 | 4.1983000 | 0.0086172 | hsa-miR-411-5p | hsa-miR-625-3p |
| 5 | hsa_circRNA_101967 | hsa_circ_0041732 | FAM64A | 3.9048000 | 0.0387100 | hsa-miR-412-3p | hsa-miR-18a-3p |
| 6 | hsa_circRNA_100834 | hsa_circ_0022392 | FADS2 | 3.8479000 | 0.0012834 | hsa-miR-873-5p | hsa-miR-23b-5p |
| 7 | hsa_circRNA_100833 | hsa_circ_0022383 | FADS2 | 3.6633000 | 0.0002395 | hsa-miR-765 | hsa-miR-495-3p |
| 8 | hsa_circRNA_104816 | hsa_circ_0087493 | IARS | 3.5465000 | 0.0464669 | hsa-miR-561-5p | hsa-miR-140-3p |
| 9 | hsa_circRNA_104575 | hsa_circ_0083766 | EPHX2 | 3.5142000 | 0.0470290 | hsa-miR-519e-5p | hsa-miR-519d-5p |
| 10 | hsa_circRNA_104852 | hsa_circ_0006174 | RAD23B | 3.3765000 | 0.0068750 | hsa-miR-138-5p | hsa-miR-325 |
| 11 | hsa_circRNA_102888 | hsa_circ_0003915 | SATB2 | 3.3296165 | 0.0035152 | hsa-miR-30c-1-3p | hsa-miR-9-5p |
| 12 | hsa_circRNA_102225 | hsa_circ_0046188 | NPLOC4 | 3.3050163 | 0.0005792 | hsa-miR-520g-3p | hsa-miR-520h |
| 13 | hsa_circRNA_104892 | hsa_circ_0002052 | PAPPA | 3.2374495 | 0.0098538 | hsa-miR-199b-5p | hsa-miR-505-3p |
| 14 | hsa_circRNA_103830 | hsa_circ_0072389 | HMGCS1 | 3.1938823 | 0.0047427 | hsa-miR-625-3p | hsa-miR-448 |
| 15 | hsa_circRNA_102885 | hsa_circ_0007422 | SATB2 | 3.1682414 | 0.0068020 | hsa-miR-328-3p | hsa-miR-301a-5p |
| 16 | hsa_circRNA_101748 | hsa_circ_0003645 | C16orf62 | 3.1109357 | 0.0287001 | hsa-miR-1301-3p | hsa-miR-422a |
| 17 | hsa_circRNA_101202 | hsa_circ_0029340 | SCARB1 | 3.0525526 | 0.0000192 | hsa-miR-508-5p | hsa-miR-378a-5p |
| 18 | hsa_circRNA_102546 | hsa_circ_0051042 | FBL | 2.8390969 | 0.0063941 | hsa-miR-520g-3p | hsa-miR-520h |
| 19 | hsa_circRNA_102227 | hsa_circ_0005221 | NPLOC4 | 2.6967122 | 0.0104648 | hsa-miR-520g-3p | hsa-miR-520h |
| 20 | hsa_circRNA_101744 | hsa_circ_0005699 | C16orf62 | 2.6752777 | 0.0436472 | hsa-miR-512-5p | hsa-miR-553 |
| 21 | hsa_circRNA_103998 | hsa_circ_0074817 | EBF1 | 2.6680746 | 0.0023879 | hsa-miR-21-3p | hsa-miR-651-3p |
| 22 | hsa_circRNA_400100 | hsa_circ_0092364 | GTF3C5 | 2.6648212 | 0.0409349 | hsa-miR-301a-5p | hsa-miR-185-3p |
| 23 | hsa_circRNA_103886 | hsa_circ_0072940 | TNPO1 | 2.6432052 | 0.0497342 | hsa-miR-578 | hsa-miR-148b-5p |
| 24 | hsa_circRNA_104940 | hsa_circ_0089153 | NUP214 | 2.6023585 | 0.0375397 | hsa-miR-608 | hsa-miR-185-3p |
| 25 | hsa_circRNA_100266 | hsa_circ_0000087 | USP33 | 2.5605774 | 0.0112954 | hsa-miR-29a-5p | hsa-miR-1323 |
| 26 | hsa_circRNA_102886 | hsa_circ_0008928 | SATB2 | 2.5462745 | 0.0323845 | hsa-miR-30c-1-3p | hsa-miR-185-5p |
| 27 | hsa_circRNA_102286 | hsa_circ_0005653 | USP14 | 2.5035855 | 0.0260828 | hsa-miR-362-5p | hsa-miR-627-3p |
| 28 | hsa_circRNA_101886 | hsa_circ_0000720 | PLCG2 | 2.4480285 | 0.0080590 | hsa-miR-578 | hsa-miR-367-5p |
| 29 | hsa_circRNA_102450 | hsa_circ_0006877 | LDLR | 2.3929836 | 0.0068749 | hsa-miR-589-5p | hsa-miR-744-5p |
| 30 | hsa_circRNA_103997 | hsa_circ_0074816 | EBF1 | 2.3604648 | 0.0122048 | hsa-miR-21-3p | hsa-miR-651-3p |
| 31 | hsa_circRNA_104983 | hsa_circ_0089974 | NHS | 2.3549442 | 0.0422526 | hsa-miR-30b-3p | hsa-miR-603 |
| 32 | hsa_circRNA_104850 | hsa_circ_0087855 | RAD23B | 2.2399567 | 0.0059905 | hsa-miR-138-5p | hsa-miR-325 |
| 33 | hsa_circRNA_103029 | hsa_circ_0059955 | ITCH | 2.1753229 | 0.0129696 | hsa-miR-578 | hsa-miR-628-5p |
| 34 | hsa_circRNA_104597 | hsa_circ_0084021 | PLEKHA2 | 2.1622322 | 0.0243433 | hsa-miR-659-3p | hsa-miR-548d-5p |
| 35 | hsa_circRNA_104851 | hsa_circ_0008812 | RAD23B | 2.1602348 | 0.0090571 | hsa-miR-138-5p | hsa-miR-325 |
| 36 | hsa_circRNA_103176 | hsa_circ_0062545 | GUSBP11 | 2.1313193 | 0.0121979 | hsa-miR-509-5p | hsa-miR-134-5p |
| 37 | hsa_circRNA_102228 | hsa_circ_0000814 | NPLOC4 | 2.0999435 | 0.0077736 | hsa-miR-511-5p | hsa-miR-764 |
| **Continued** | | | | | | | |
| **Rank** | **circRNA** | **Alias** | **Gene Symbol** | **FC** | ***P*-value** | **MRE** | |
| 38 | hsa_circRNA_100655 | hsa_circ_0006371 | LCOR | 2.0913011 | 0.0411269 | hsa-miR-152-5p | hsa-miR-329-5p |
| 39 | hsa_circRNA_103831 | hsa_circ_0072391 | HMGCS1 | 2.0587619 | 0.0191453 | hsa-miR-625-3p | hsa-miR-129-5p |
| 40 | hsa_circRNA_103244 | hsa_circ_0008806 | CCDC134 | 2.0422248 | 0.0452112 | hsa-miR-625-5p | hsa-miR-766-3p |
| 41 | hsa_circRNA_104056 | hsa_circ_0007429 | RREB1 | 2.0420237 | 0.0016341 | hsa-miR-637 | hsa-miR-623 |
| 42 | hsa_circRNA_103871 | hsa_circ_0004837 | SMA4 | 2.0316373 | 0.0273425 | hsa-miR-92a-2-5p | hsa-miR-377-5p |
| 43 | hsa_circRNA_102080 | hsa_circ_0043921 | AARSD1 | 2.0274256 | 0.0362170 | hsa-miR-188-3p | hsa-miR-619-5p |
| 44 | hsa_circRNA_102451 | hsa_circ_0003892 | LDLR | 2.0249474 | 0.0264343 | hsa-miR-608 | hsa-miR-134-3p |
| 45 | hsa_circRNA_102949 | hsa_circ_0058792 | AGAP1 | 2.0177376 | 0.0337605 | hsa-miR-431-3p | hsa-miR-181c-5p |
| 46 | hsa_circRNA_103837 | hsa_circ_0004840 | ITGA1 | 2.0085074 | 0.0185150 | hsa-miR-145-5p | hsa-miR-429 |
| **Down-regulated Differentiation (AA VS YA)** | | | | | | | |
| 1 | hsa_circRNA_101889 | hsa_circ_0040705 | USP10 | 6.6744000 | 0.0242806 | hsa-miR-670-3p | hsa-miR-103a-2-5p |
| 2 | hsa_circRNA_102273 | hsa_circ_0046600 | B3GNTL1 | 5.4184000 | 0.0301344 | hsa-miR-518a-5p | hsa-miR-527 |
| 3 | hsa_circRNA_100332 | hsa_circ_0014130 | PIP5K1A | 4.2928000 | 0.0343650 | hsa-miR-892a | hsa-miR-216a-3p |
| 4 | hsa_circRNA_103611 | hsa_circ_0069323 | GPR125 | 3.2502000 | 0.0473540 | hsa-miR-485-5p | hsa-miR-140-3p |
| 5 | hsa_circRNA_104379 | hsa_circ_0080251 | GBAS | 2.5311000 | 0.0468073 | hsa-miR-185-3p | hsa-miR-96-5p |
| 6 | hsa_circRNA_102660 | hsa_circ_0053422 | SLC30A6 | 2.4374000 | 0.0032883 | hsa-miR-141-5p | hsa-miR-449c-5p |
| 7 | hsa_circRNA_103612 | hsa_circ_0069338 | SEPSECS | 2.0951000 | 0.0039483 | hsa-miR-649 | hsa-miR-9-5p |
| 8 | hsa_circRNA_104457 | hsa_circ_0003331 | PUS7 | 2.0716000 | 0.0417426 | hsa-miR-599 | hsa-miR-197-5p |
| 9 | hsa_circRNA_104755 | hsa_circ_0086686 | UBAP2 | 2.0534000 | 0.0123885 | hsa-miR-512-3p | hsa-miR-370-3p |
| 10 | hsa_circRNA_101852 | hsa_circ_0040039 | SNTB2 | 2.0500000 | 0.0116944 | hsa-miR-424-5p | hsa-miR-15b-5p |
| 11 | hsa_circRNA_101562 | hsa_circ_0035943 | DENND4A | 2.0068164 | 0.0093610 | hsa-miR-641 | hsa-miR-199b-3p |
| FC, fold change; MRE, miRNA response elements. | | | | | | | |
