# Supplementary material for: Circular RNA expression profiling of human granulosa cells during maternal aging reveals novel transcripts associated with assisted reproductive technology outcomes
Source: PLoS One. 2017 Jun 23;12(6):e0177888. doi: 10.1371/journal.pone.0177888 (PMC5482436; doi:10.1371/journal.pone.0177888)
Supplement: S5 Table — (DOCX) [file pone.0177888.s010.docx]

| **S5 Table. The correlation coefficients (r) of circRNAs and clinical variables (n=80) were analyzed by Spearman.** | | | | | | | | | |
| --- | --- | --- | --- | --- | --- | --- | --- | --- | --- |
| **circRNAs** | **Statistics** | **BMI (kg/m^2^)** | **FSH (IU/l)** | **LH (IU/l)** | **E2 (pg/ml)** | **T (ng/dl)** | **AMH (ng/ml)** | **AFC (n)** | **Peak E2 (pg/ml)** |
| **circRNA_103827** | r | .000 | .205 | .005 | -.020 | .054 | **-.283^*^** | **-.305^**^** | **-.325^**^** |
|  | *P* | 1.000 | .068 | .966 | .859 | .632 | .011 | .006 | .003 |
| **circRNA_104816** | r | .065 | **.265^*^** | .094 | **-.259^*^** | -.143 | **-.311^*^** | **-.314^**^** | **-.342^**^** |
|  | *P* | .567 | .018 | .408 | .020 | .206 | .005 | .005 | .002 |
