# Supplementary material for: Circular RNA expression profiling of human granulosa cells during maternal aging reveals novel transcripts associated with assisted reproductive technology outcomes
Source: PLoS One. 2017 Jun 23;12(6):e0177888. doi: 10.1371/journal.pone.0177888 (PMC5482436; doi:10.1371/journal.pone.0177888)
Supplement: S6 Table — (DOCX) [file pone.0177888.s011.docx]

| **S6 Table. The correlation coefficients (r) of circRNAs and ART outcomes (n=80) were analyzed by Spearman.** | | | | | | | | | |
| --- | --- | --- | --- | --- | --- | --- | --- | --- | --- |
| **circRNAs** | **Statistics** | **Retrieved oocytes** | **MII oocytes** | **2PN** | **Embryos**  **< 10% fragments** | **Top quality embryos** | | **Clinical pregnancy** | **Live birth** |
| **circRNA_103827** | r | **-.345^**^** | **-.313^*^** | **-.274^*^** | **-.253^*^** | | **-.235^*^** | **-.380^**^** | **-.310^**^** |
|  | *P* | .002 | .005 | .014 | .026 | | .036 | .001 | .005 |
| **circRNA_104816** | r | **-.294^**^** | **-.268^*^** | **-.269^*^** | **-.344^**^** | | **-.221^*^** | **-.291^**^** | **-.228^*^** |
|  | *P* | .008 | .016 | .016 | .003 | | .049 | .009 | .042 |
