# Supplementary material for: Circular RNA expression profiling of human granulosa cells during maternal aging reveals novel transcripts associated with assisted reproductive technology outcomes
Source: PLoS One. 2017 Jun 23;12(6):e0177888. doi: 10.1371/journal.pone.0177888 (PMC5482436; doi:10.1371/journal.pone.0177888)
Supplement: S7 Table — r-FSH, recombinant follicle-stimulating hormone; HP-hMG, highly purified human menopausal gonadotropin; E2,17β-estradiol. * P < 0.05; ** P < 0.01. (DOCX) [file pone.0177888.s012.docx]

| **S7 Table.** **CircRNA_103827 and circRNA_104816 expression levels in granulosa cells relative to ovarian stimulation protocols.** | | | | | | | | |
| --- | --- | --- | --- | --- | --- | --- | --- | --- |
| **Variable** | | **Mean ± SD** | **Min-Max** | **N** | **circRNA_103827** | ***P*-value** | **circRNA_104816** | ***P*-value** |
|  |  |  |  | (total=80) | Mean ± SD |  | Mean ± SD |  |
| Agonist protocol | | - | - | 48 | 4.85 ± 7.26 | 0.317 | 4.06 ± 8.54 | 0.609 |
| Antagonist protocol | | - | - | 25 | 3.57 ± 5.12 |  | 3.83 ± 9.26 |  |
| Days of stimulation |  | 10.1 ± 2.6 | 6-17 |  |  |  |  |  |
| ≤ 10 | | - | - | 59 | 4.69 ± 6.45 | 0.956 | 4.98 ± 10.54 | 0.983 |
| > 10 | | - | - | 21 | 3.69 ± 4.62 |  | 3.47 ± 6.97 |  |
| The type of gonadotropins | |  |  |  |  |  |  |  |
| r-FSH | | - | - | 23 | 4.24 ± 5.78 | 0.083 | 3.73 ± 9.29 | 0.076 |
| HP-hMG | | - | - | 20 | 6.95 ± 8.09 |  | 6.54 ± 11.46 |  |
| r-FSH + HP-hMG | | - | - | 37 | 3.18 ± 4.36 |  | 4.05 ± 9.05 |  |
| Total dose of gonadotropins (IU/l) | | 2094 ± 870 | 670-4725 |  |  |  |  |  |
| < 2500 | | - | - | 63 | 4.24 ± 6.22 | 0.240 | 4.75 ± 10.24 | 0.529 |
| ≥2500 | | - | - | 17 | 5.12 ± 5.27 |  | 3.97 ± 7.63 |  |
| Hormonal levels at oocyte pick up | |  |  |  |  |  |  |  |
| Peak E2 (pg/ml) |  | 2270 ± 1605 | 264-5376 |  |  |  |  |  |
| < 2000 | | - | - | 37 | 6.35 ± 7.68 | **0.006*** | 6.59 ± 11.70 | **0.004**** |
| ≥ 2000 | | - | - | 43 | 2.77 ± 3.36 |  | 2.85 ± 7.30 |  |
| Progesterone (ng/ml) |  | 0.92 ± 0.44 | 0.1-2.2 |  |  |  |  |  |
| < 1 | | - | - | 50 | 4.53 ± 5.81 | 0.777 | 4.46 ± 8.76 | 0.407 |
| ≥ 1 | | - | - | 30 | 4.27 ± 6.43 |  | 4.79 ± 11.26 |  |
| r-FSH, recombinant follicle-stimulating hormone; HP-hMG, highly purified human menopausal gonadotropin; E2,17β-estradiol.  * *P* < 0.05; ** *P* < 0.01. | | | | | | | | |
